# Association Between Cancer Incidence and Mortality in Web-Based Data in China: Infodemiology Study

**DOI:** 10.2196/10677

**Published:** 2019-01-29

**Authors:** Chenjie Xu, Yi Wang, Hongxi Yang, Jie Hou, Li Sun, Xinyu Zhang, Xinxi Cao, Yabing Hou, Lan Wang, Qiliang Cai, Yaogang Wang

**Affiliations:** 1 School of Public Health Tianjin Medical University Tianjin China; 2 Tandon School of Engineering New York University New York, NY United States; 3 School of Basic Medical Sciences Tianjin Medical University Tianjin China; 4 School of Nursing Tianjin Medical University Tianjin China; 5 The Second Hospital of Tianjin Medical University Tianjin Medical University Tianjin China

**Keywords:** cancer, incidence, mortality, web-based data, internet searching

## Abstract

**Background:**

Cancer poses a serious threat to the health of Chinese people, resulting in a major challenge for public health work. Today, people can obtain relevant information from not only medical workers in hospitals, but also the internet in any place in real-time. Search behaviors can reflect a population’s awareness of cancer from a completely new perspective, which could be driven by the underlying cancer epidemiology. However, such Web-retrieved data are not yet well validated or understood.

**Objective:**

This study aimed to explore whether a correlation exists between the incidence and mortality of cancers and normalized internet search volumes on the big data platform, Baidu. We also assessed whether the distribution of people who searched for specific types of cancer differed by gender. Finally, we determined whether there were regional disparities among people who searched the Web for cancer-related information.

**Methods:**

Standard Boolean operators were used to choose search terms for each type of cancer. Spearman’s correlation analysis was used to explore correlations among monthly search index values for each cancer type and their monthly incidence and mortality rates. We conducted cointegration analysis between search index data and incidence rates to examine whether a stable equilibrium existed between them. We also conducted cointegration analysis between search index data and mortality data.

**Results:**

The monthly Baidu index was significantly correlated with cancer incidence rates for 26 of 28 cancers in China (lung cancer: *r*=.80, *P*<.001; liver cancer: *r*=.28, *P*=.016; stomach cancer: *r*=.50, *P*<.001; esophageal cancer: *r*=.50, *P*<.001; colorectal cancer: *r*=.81, *P*<.001; pancreatic cancer: *r*=.86, *P*<.001; breast cancer: *r*=.56, *P*<.001; brain and nervous system cancer: *r*=.63, *P*<.001; leukemia: *r*=.75, *P*<.001; Non-Hodgkin lymphoma: *r*=.88, *P*<.001; Hodgkin lymphoma: *r*=.91, *P*<.001; cervical cancer: *r*=.64, *P*<.001; prostate cancer: *r*=.67, *P*<.001; bladder cancer: *r*=.62, *P*<.001; gallbladder and biliary tract cancer: *r*=.88, *P*<.001; lip and oral cavity cancer: *r*=.88, *P*<.001; ovarian cancer: *r*=.58, *P*<.001; larynx cancer: *r*=.82, *P*<.001; kidney cancer: *r*=.73, *P*<.001; squamous cell carcinoma: *r*=.94, *P*<.001; multiple myeloma: *r*=.84, *P*<.001; thyroid cancer: *r*=.77, *P*<.001; malignant skin melanoma: *r*=.55, *P*<.001; mesothelioma: *r*=.79, *P*<.001; testicular cancer: *r*=.57, *P*<.001; basal cell carcinoma: *r*=.83, *P*<.001). The monthly Baidu index was significantly correlated with cancer mortality rates for 24 of 27 cancers. In terms of the whole population, the number of women who searched for cancer-related information has slowly risen over time. People aged 30-39 years were most likely to use search engines to retrieve cancer-related knowledge. East China had the highest Web search volumes for cancer.

**Conclusions:**

Search behaviors indeed reflect public awareness of cancer from a different angle. Research on internet search behaviors could present an innovative and timely way to monitor and estimate cancer incidence and mortality rates, especially for cancers not included in national registries.

## Introduction

**Figure 1:**
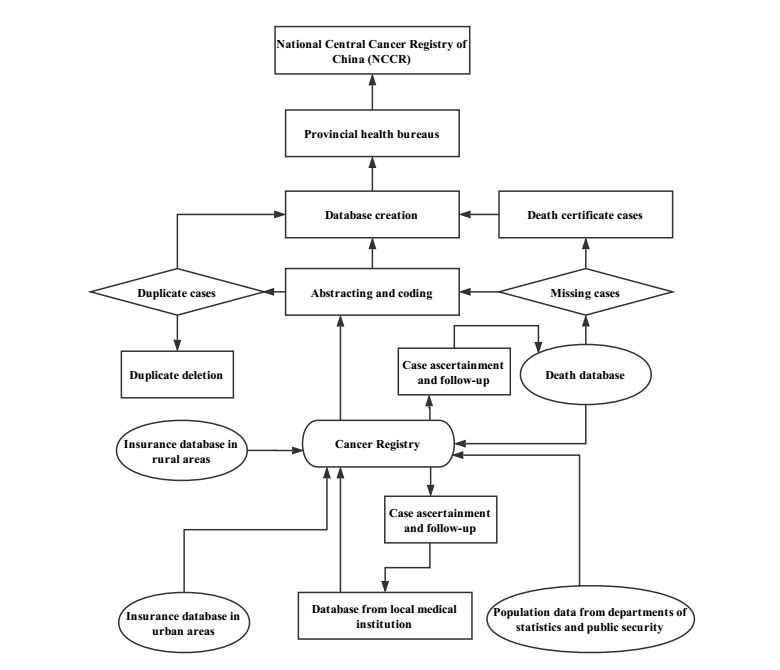
Flow diagram of the cancer registration system.

Cancer affects people of all socioeconomic levels all over the world [1,2]. The global burden of cancer is increasing [3]. The population of China accounts for 19.3% of the global population, and the incidence of cancer accounts for 22% of global cancer incidence, ranking first in the world. Cancer deaths in China account for about 27% of global cancer deaths. Cancer mortality in China is also higher than the global average of 17% [4,5]. There is a growing demand for knowledge about cancers, but the registration of cancer cases in China requires complicated procedures (Figure 1) [6]. Traditional epidemiologic methods usually have a 3-year delay until incidence and mortality data are publicly reported due to the time required for data collection, compilation, quality control, and dissemination [7-9]. However, because nonmelanoma skin cancers (basal cell carcinoma and squamous cell carcinoma) are relatively nonlethal and curable by surgery, they are not covered by national surveillance and lack corresponding epidemiological data [10]. Given the inadequacy of traditional methods and the absence of data sources, internet search data can be used to estimate the characteristics of diseases [11].

Today, social media and medical forums are rapidly spreading, and internet users are increasingly exchanging health-related information. When people feel ill or have early symptoms, they may tend to first look for relevant health information on the internet for self-assessment. Some studies have improved the surveillance of epidemics and examined public interest in multiple health topics by monitoring the search behaviors of millions of users and conducting data mining through Google [12]. Other studies have tried to identify medication concerns, examine patient experience sentiments, and understand public perceptions by text mining social network data (eg, Facebook and Twitter) [13,14]. Studies have found that about 63% of cancer patients use the internet to retrieve cancer-related information [15-17]. A significant number of cancer patients utilize the internet to collect information about their respective diagnoses. A substantial number of cancer patients utilize the internet to gather information about their course of disease development [18]. It is becoming increasingly clear that the internet is a frequent source of information in our society for patients with cancer [19]. Compared with the past, at present, more Chinese internet users choose to retrieve information from the internet to obtain diagnosis and treatment information [20]. In addition to the patients themselves, friends and family members look for disease information using search engines, apps, and other resources, which are often designed to provide potentially helpful suggestions [21]. Studies in the United States have found a positive correlation between Google search volume and cancer incidence and mortality [22,23]. With the advancement of methodologies using “Big Data,” researchers are able to track diseases by the use of common internet search engines as a real-time tool [24]. Web search content is publicly available worldwide, providing valuable data for research, including health-related topics [25].

In this study, we tracked and monitored the Baidu index [26] and the search behaviors of Chinese internet users to explore public search interest in cancers. We also explored whether gender, age, and regional differences existed in search behaviors. We hypothesized that internet search volumes can reflect the disease characteristics of cancer (such as incidence and mortality) and provide an additional means of cancer surveillance in China.

## Methods

### Cancer Data

National-level incidence and mortality rates of cancers in China were obtained for the period 2011-2016 from the Global Burden of Disease database, which is publicly available [27]. For this study, we selected 28 types of cancer, including lung cancer, liver cancer, stomach cancer, esophageal cancer, colon and rectal cancer, pancreatic cancer, breast cancer, brain and nervous system cancer, cervical cancer, prostate cancer, nasopharynx cancer, bladder cancer, gallbladder and biliary tract cancer, lip and oral cavity cancer, ovarian cancer, larynx cancer, kidney cancer, testicular cancer, uterine cancer, thyroid cancer, multiple myeloma, leukemia, Non-Hodgkin lymphoma, malignant skin melanoma, Hodgkin lymphoma, mesothelioma, basal cell carcinoma, and squamous cell carcinoma. We also obtained the incidence and mortality rates of cancers according to gender, although mortality rates for basal cell carcinoma were missing. Due to the lack of monthly data for incidence and mortality rates, we used annual incidence and mortality rates for each cancer instead.

### Web Search Data

This study mainly considered cancer search index values from Chinese search engines. The Baidu index was used as the entry point to launch the corresponding research [26]. Among Chinese search engine users (searches are usually conducted in Chinese), Baidu accounts for 92.1% of searches, followed by Haosou [28] and Sougou [29]. Regarding mobile search engines, the brand performance is the same: Baidu ranks first with 93.1% of the use rate. Baidu is a well-known Chinese search engine with powerful real-time functions [30]; it holds a strong position in China. Baidu is a very large information resource-sharing platform that Chinese netizens depend on. The Baidu index has proven to be a useful indicator of public interest in and awareness of health-related topics [31,32]. We assumed the Baidu index could best represent the retrieval preferences of Chinese internet users.

The Baidu index derives from search frequencies on the Baidu search engine; it is calculated and displayed based on the search volumes of specific keywords entered by users [33]. We entered the search terms for cancers according to the settings in the Baidu index to obtain the monthly total search index values of all cancers from January 2011 to December 2016. The daily Baidu index is the weighted sum of the search frequency for a keyword based on its daily search volume on Baidu. The monthly Baidu search index value is the average of the total daily search index values in a month. We also obtained gender, age, and regional distribution data for people who retrieved cancer information online.

### Search Terms

In this study, cancer awareness was examined on the basis of the general population’s ability to seek information on or pay attention to the disease. Because Baidu is a Chinese search engine, the search terms are all expressed in Chinese characters. Given the diverse meanings of Chinese characters, in addition to their formal Chinese names, some cancers have various synonyms. All their formal Chinese names were referenced to the International Classification of Diseases for Oncology. Therefore, we selected both the formal Chinese names and common terms for various cancers while searching. Standard Boolean operators were used to combine terms. The search index value for each cancer could be incorporated into five keywords, and the selected terms were not searched in quotes. For most cancers, we used two or more search terms in Chinese to cover as many synonyms as possible.

The Baidu index covers the function of keyword analysis, which is the process of scientifically determining keywords based on the mode through which the searchers initiate a search request. According to the time period of the research (January 2011 to December 2016), the Baidu index system automatically analyzed the flow and trend of keywords imported in the Baidu search engine. We first entered the formal Chinese names of various cancers as keywords. The keyword analysis function automatically generated a corresponding number of related words and the search demand of the related words themselves. These words could be used as search terms to reflect people’s retrieval needs. The function of keyword analysis helped us screen the search terms preliminary. We also conducted different retrieval methods for keyword selection to make the process more rigorous. We conducted comparative retrieval, cumulative retrieval, and combined retrieval for keywords and related words. Comparative retrieval aims to separate different keywords with commas among multiple words, which can realize the comparative query of keyword data. Cumulative retrieval indicates that among different keywords, different keywords are connected by a plus sign, and the addition of different keyword data can be realized. The aggregated data are presented as a combination of keywords. Combined retrieval is a combination of “comparative retrieval” and “cumulative retrieval.” Subsequently, the search terms of each cancer can be determined.

For non-Hodgkin lymphoma, we also added the more common term “lymphoma” because that search term is twice as common as “non-Hodgkin lymphoma,” and approximately 90% of lymphomas are non-Hodgkin lymphoma [34,35]. For prostate cancer, larynx cancer, Hodgkin lymphoma, and mesothelioma, we only used one search term, since their synonyms were not included in the Baidu index. We were unable to include their synonyms because they lacked unifying search terms with adequate search data for the analysis. Multimedia Appendix 1 shows all the search terms.

### Statistical Analysis

First, we performed the Spearman correlation analysis to evaluate the relationship between the known cancer incidence and mortality rates for all cancer types and the Baidu index for the period 2011-2016. The distribution of the original variables is not required in the Spearman correlation analysis, as it is a nonparametric statistical method, and the scope of application is wider; thus, statistical significance was set as .05 (two-sided test).

Second, we used the Engel-Grange test to determine whether there was cointegration or long-term association between the three indicators. We defined the search index values and the incidence and mortality rates for each type of cancer over the past 6 years as time-series data. To eliminate heteroscedasticity in the time series, in the first step, we obtained the log version of the Baidu index, and incidence and mortality rates [36]. The advantage of this step is that data with large spacing can be converted into data with small spacing. Thereafter, we used unit root tests to examine whether the time series of the Baidu index for cancer searches and the time series for cancer incidence and mortality rates were stationary. If the three time series were all stationary at the same level, we estimated cointegration using ordinary least squares. We performed two types of cointegration analysis. Baidu index for cancer searches was used as the independent variable. The cancer incidence rate and cancer mortality rate were used as the dependent variable separately. In the third step, we used unit root test to test whether the residual series of the cointegrating regression model was stationary, which would show that the time series variables were cointegrated.

Statistical analysis was conducted using IBM SPSS (version 22.0, IBM Corporation, Armonk, NY), EViews (version 8, IHS Global Inc, London, United Kingdom), and R project (version 3.4, R Development Core Team, Vienna, Austria). We used Tableau (version 2018.3, Tableau Software, Seattle, WA) to conduct statistical analysis and create figures.

## Results

### Incidence and Mortality Rates of Cancers in China

**Figure 2:**
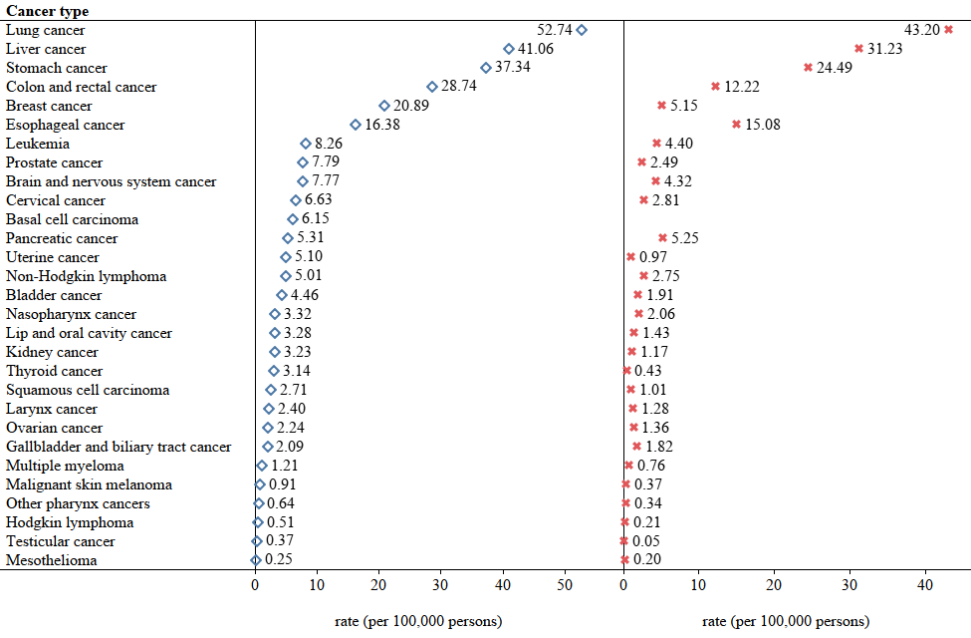
Incidence and mortality rates of cancers in China, 2016. Data were obtained from the Global Burden of Disease database. The blue signs represent incidence rates and the red signs represent mortality rates.

**Figure 3:**
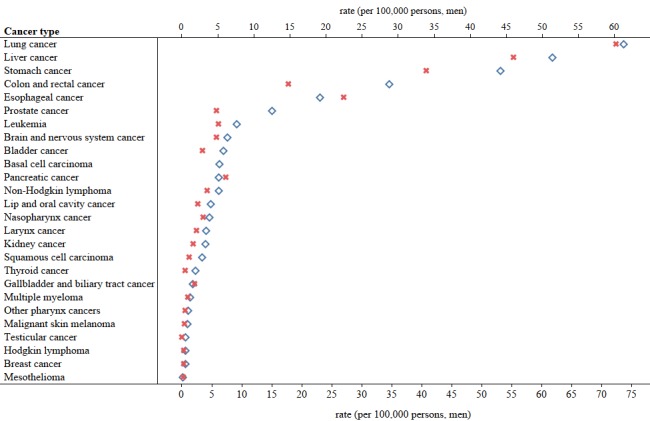
Incidence and mortality rates of cancers in China among men in 2016. The blue signs represent incidence rates and the red signs represent mortality rates.

**Figure 4:**
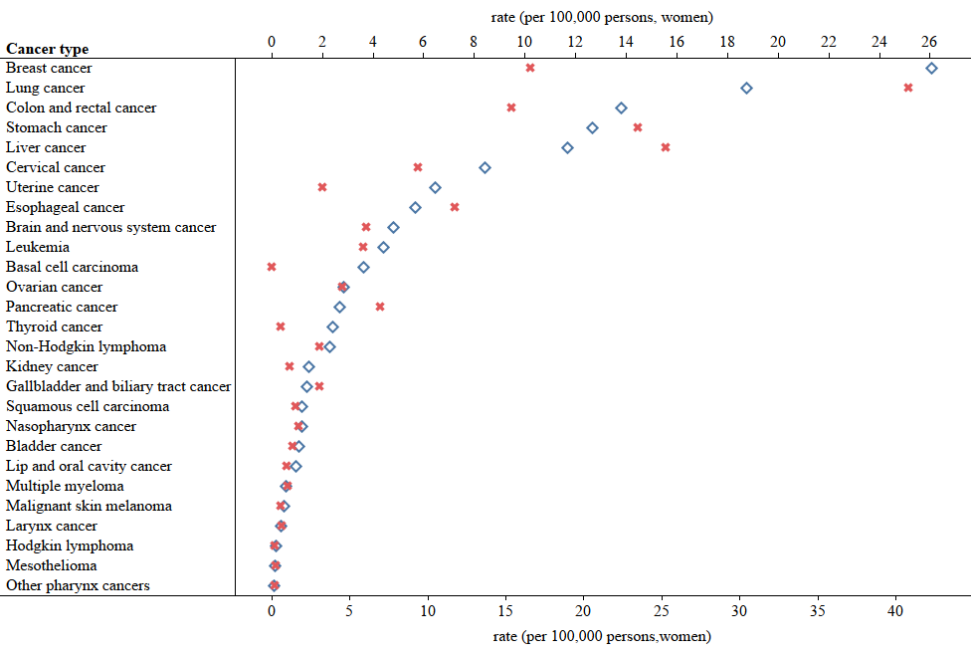
Incidence and mortality rates of cancers in China among women in 2016. The blue signs represent incidence rates and the red signs represent mortality rates.

We obtained the cancer incidence and mortality rates from 2011 to 2016 using data from the Global Burden of Disease database [27]. We illustrated the differences in the incidence and mortality rates of 28 cancers in China as well as the differences between men and women in 2016 (Figures 2, 3, and 4).

### Trends in Web-Based Data, Cancer Incidence, and Mortality Rates

**Figure 5:**
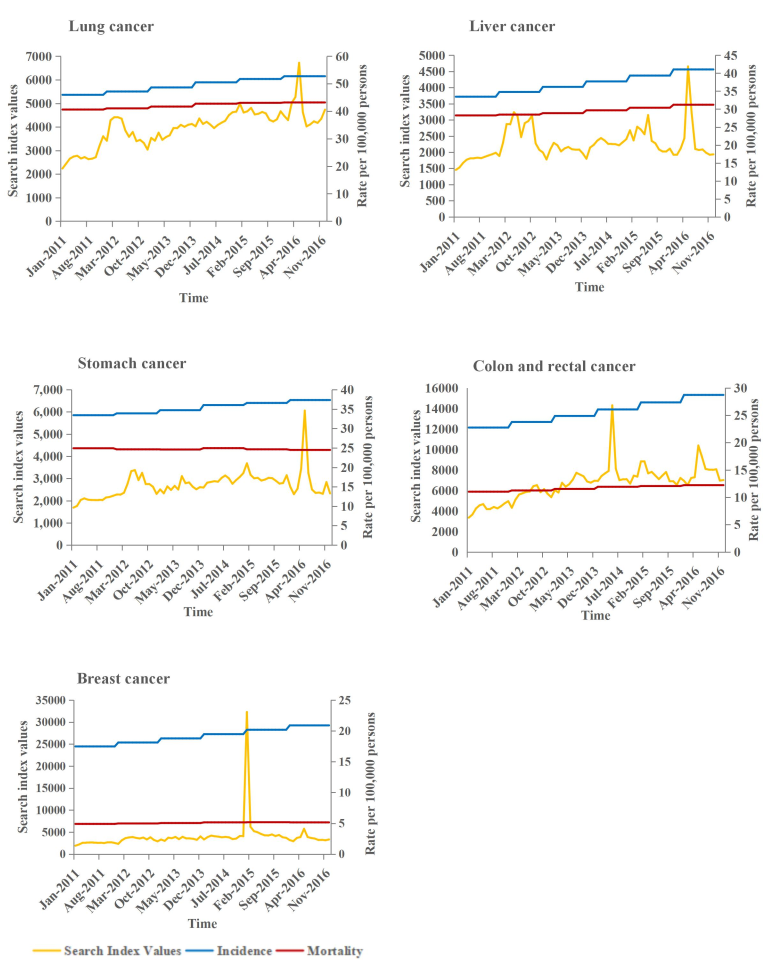
Time series of search index values and incidence and mortality rates (top five most commonly occurring cancers).

Figure 5 shows a times series of the Baidu index and the incidence and mortality rates for the top five most common cancers in China. The remaining cancer types are shown in the Multimedia Appendix 2. The search index for these cancers is relatively flat at first, eventually showing a fluctuating trend over time. For multiple myeloma, part of the figure shows a “W” shape during this time, indicating significant fluctuation in the search index values. For mesothelioma, part of the figure shows a “V” shape, representing one search valley. For malignant skin melanoma, the monthly Baidu search index values showed a downward trend at first. From January 2015 to April 2016, the search trends of non-Hodgkin lymphoma and Hodgkin lymphoma showed consistent changes in volatility. Overall, searches for cancer terms showed an upward trend. At the same time, single or multiple peaks emerged in the fluctuation. The Baidu index data for breast cancer reached a peak in 2015; the search index value reached a maximum of 32,284 and the search frequency suddenly increased dramatically. A similar trend was observed for testicular cancer in November 2016, although the average search index was high. The trend values for uncommon cancers were relatively volatile.

### Gender Differences

**Figure 6:**
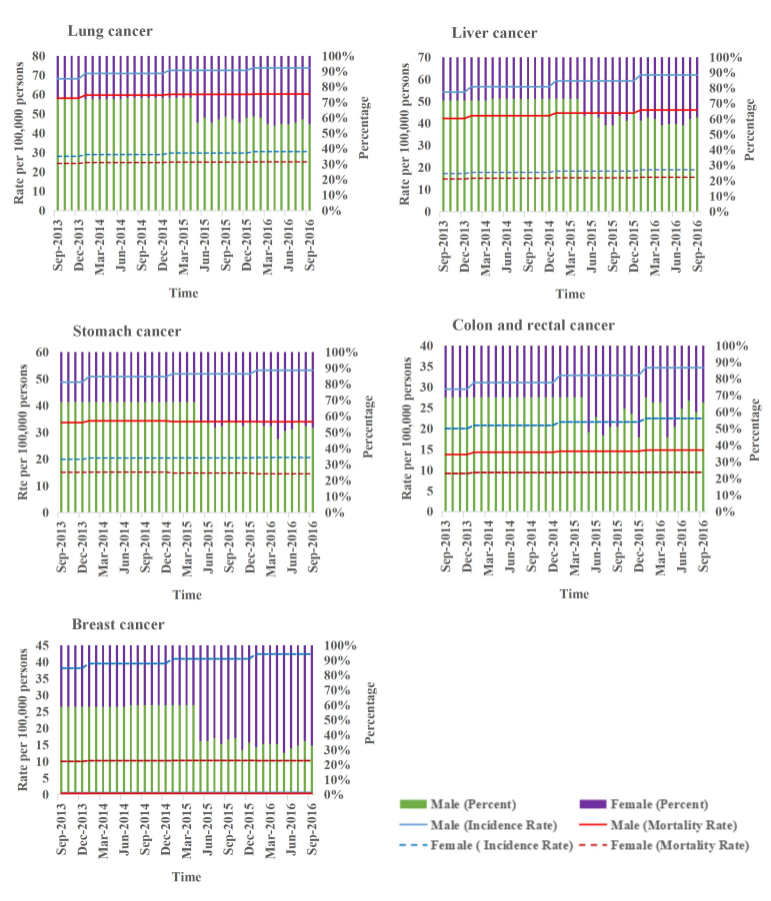
Incidence, mortality, and search distribution of cancers divided by gender (top five most commonly occurring cancers). The percentile chart represents the change from September 2013 to September 2016.

For Hodgkin lymphoma, gender percentage data were missing before May 2015. The incidence and mortality rates of brain, nervous system, thyroid, gallbladder, and biliary tract cancers were found to be higher for women than for men. Incidence and mortality rates of female-specific cancers (breast, cervical, and uterine cancers) were also higher than those of male-specific cancers (prostate and testicular). Other cancers had higher incidence and mortality rates among men than among women. Incidence and mortality rates have increased annually among both men and female for lung cancer, liver cancer, colorectal cancer, pancreatic cancer, brain and nervous system cancer, non-Hodgkin lymphoma, prostate cancer, bladder cancer, gallbladder and biliary tract cancer, lip and oral cavity cancer, ovarian cancer, kidney cancer, multiple myeloma, and malignant skin melanoma. Relatively, men paid more attention to search terms related to these cancers than women. In terms of the whole population, the number of women who searched for cancer-related information has slowly risen since 2015, while the number of men has shown a downward trend. This trend is even more obvious for female-specific cancers such as breast, cervical, ovarian, and uterine cancers. Initially, more men searched for terms related to breast cancer, but over time, an increasing number of women searched for such terms. More men paid attention to prostate and testicular cancers than women (Figure 6, Multimedia Appendix 2).

### Age Distribution

**Figure 7:**
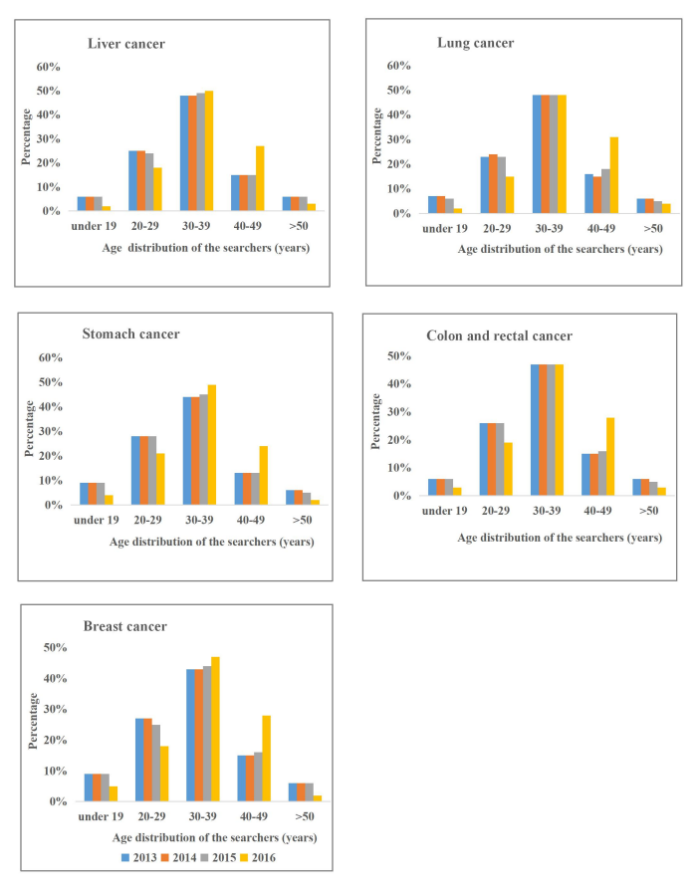
Age distribution of the searchers from 2013 to 2016 (top five most commonly occurring cancers).

Figure 7 and Multimedia Appendix 2 show the age distribution for cancer-related searches from 2013 to 2016 in China. As Figure 7 shows, the age group of 30-39 years was the largest search group for each cancer type, and the group aged over 50 years was the smallest. The proportion of the age groups of 30-39 years and 40-49 years increased over the last 4 years of the study period. The remaining age groups showed an opposite trend.

### Regional Distribution

**Figure 8:**
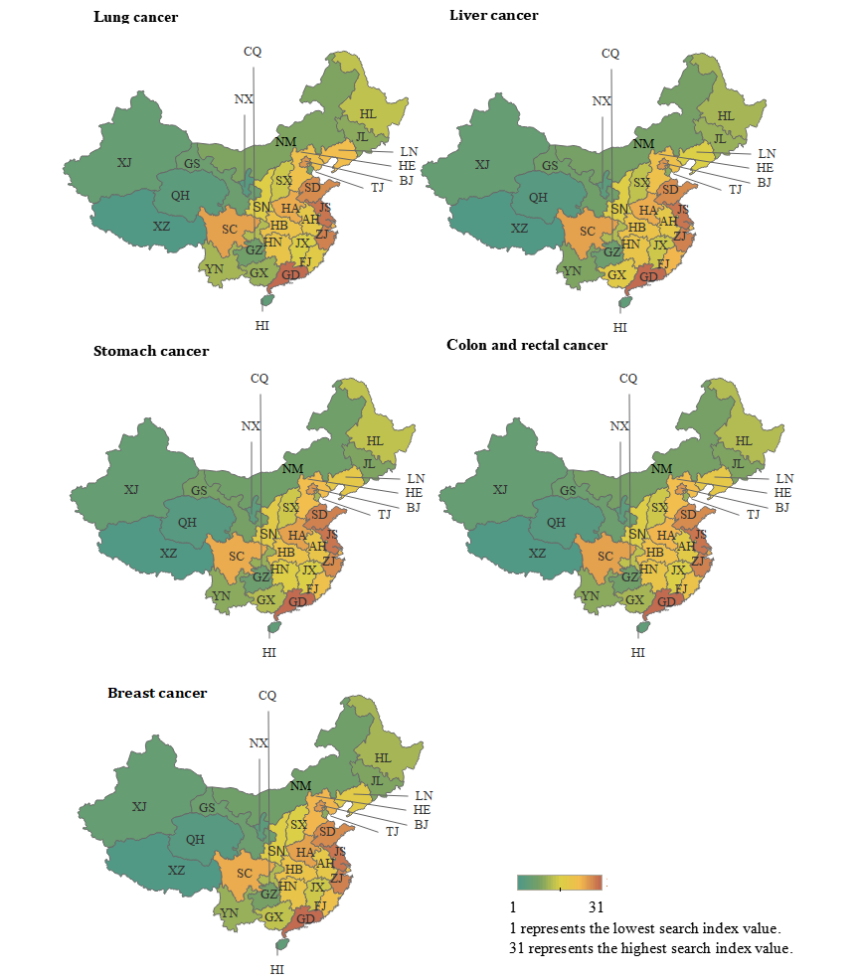
Ranking of regional distribution of the online searchers from 2013 to 2016 (top five most commonly occurring cancers) in mainland China. AH: Anhui, BJ: Beijing, FJ: Fujian, GS: Gansu, GD: Guangdong, GX: Guangxi, GZ: Guizhou, HI: Hainan, HE: Hebei, HA: Henan, HL: Heilongjiang, HB: Hubei, HN: Hunan, JL: Jilin, JS: Jiangsu, JX: Jiangxi, LN: Liaoning, NM: Inner Mongoria, NX: Ningxia, QH: Qinghai, SD: Shandong, SX: Shanxi, SN: Shaanxi, SH: Shanghai, SC: Sichuan, TJ: Tianjing, XZ: Tibet, XJ: Xinjiang, YN: Yunnan, ZJ: Zhejiang, CQ: Chongqing.

Figure 8 shows the rankings of regional cancer search index values from 2013 to 2016 in 31 Chinese provinces and cities. Ranking was determined by the size of the web search volumes. In the heat maps, Guangdong Province shows the highest search index value and Tibet shows the lowest. For the top five cancer types, search values were the highest in eastern China (Shanghai, Jiangsu Province, Zhejiang Province, Anhui Province, Fujian Province, Jiangxi Province, and Shandong Province) and the lowest in northwestern China (Shaanxi Province, Gansu Province, Qinghai Province, the Ningxia Hui autonomous region, and the Xinjiang Uygur autonomous region). North, south, central, southwest, and northeast China were ranked second to sixth, respectively, in the search index values.

**Table 1:** Correlation coefficients between search index values, incidence rate of cancers, and mortality rate of cancers.

Cancer	Correlation between search index values and incidence rate		Correlation between search index values and mortality rate	
	*r* (correlation coefficient)	*P* value	*r* (correlation coefficient)	*P* value
Lung cancer	0.80	<.001	0.80	<.001
Liver cancer	0.28	.02	0.28	.02
Stomach cancer	0.50	<.001	0.02	.88
Esophageal cancer	0.50	<.001	0.21	.08
Colon and rectal cancer	0.81	<.001	0.81	<.001
Pancreatic cancer	0.86	<.001	0.86	<.001
Breast cancer	0.56	<.001	0.76	<.001
Leukemia	0.75	<.001	-0.70	<.001
Brain and nervous system cancer	0.63	<.001	0.63	<.001
Cervical cancer	0.64	<.001	0.65	<.001
Non-Hodgkin lymphoma	0.88	<.001	0.88	<.001
Prostate cancer	0.67	<.001	0.67	<.001
Nasopharynx cancer	0.08	.51	0.44	<.001
Bladder cancer	0.62	<.001	0.62	<.001
Gallbladder and biliary tract cancer	0.88	<.001	0.88	<.001
Lip and oral cavity cancer	0.88	<.001	0.88	<.001
Ovarian cancer	0.58	<.001	0.58	<.001
Larynx cancer	0.82	<.001	0.74	<.001
Kidney cancer	0.73	<.001	0.73	<.001
Squamous cell carcinoma	0.94	<.001	0.87	<.001
Uterine cancer	0.04	.73	-0.42	<.001
Multiple myeloma	0.84	<.001	0.84	<.001
Thyroid cancer	0.77	<.001	0.77	<.001
Malignant skin melanoma	0.55	<.001	0.55	<.001
Hodgkin lymphoma	0.91	<.001	-0.91	<.001
Mesothelioma	0.79	<.001	0.79	<.001
Testicular cancer	0.57	<.001	-0.08	.48
Basal cell carcinoma	0.83	<.001	—^a^	—^a^

^a^Not available.

### Correlation Analysis

Table 1 shows the correlation coefficients between actual incidence rates and the relative Baidu index for 28 cancers in China. We found statistically significant correlations between incidence rates and the relative Baidu index for 26 cancers (nasopharynx cancer and uterine cancer did not show such correlations).

Table 1 also shows the correlation coefficients between actual mortality rates and the relative Baidu index for these cancers. Stomach cancer, esophageal cancer, and testicular cancer did not show statistically significant correlations with mortality rates. For leukemia, uterine cancer, and Hodgkin lymphoma, the relative Baidu index was negatively correlated with cancer mortality rates.

### Cointegration Analysis

Augmented Dickey-Fuller unit root test was used to examine the stationarity of the time series. Schwarz information criterion was used to determine lag length automatically. We first made logarithmic changes to the three indexes. After transformation, the series were all stationary at the first difference (Multimedia Appendix 3). Since the series were found to be stationary at the same level, the three variables satisfied the precondition of cointegration and were checked for a long-term cointegration relationship. The result of the cointegration (Engle-Granger) test showed cointegration between variables for the Baidu index and incidence rates at the first difference (Multimedia Appendix 4). The cointegration test also showed cointegration between variables for the Baidu index and mortality rates at the first difference (Multimedia Appendix 5).

## Discussion

### Principal Findings

For most cancers, the Baidu index was positively correlated with cancer incidence rates. For several cancers including lung cancer, liver cancer, stomach cancer, colon and rectal cancer, breast cancer, prostate cancer, brain and nervous system cancer, cervical cancer, pancreatic cancer, non-Hodgkin lymphoma, bladder cancer, nasopharynx cancer, lip and oral cavity cancer, kidney cancer, thyroid cancer, squamous cell carcinoma, larynx cancer, ovarian cancer, gallbladder and biliary tract cancer, multiple myeloma, malignant skin melanoma and mesothelioma, the Baidu index was positively correlated with cancer mortality rates. The results suggest that the search engine data can reflect actual prevalence to some extent. Such data sources might be particularly useful when real-time information is required or missing (eg, mortality rate of basal cell carcinoma is lacking), considering that there is often a lag of several years in the publication of cancer registration data. The results of this study suggest that we should study and make use of Web-based data and publicly available information regarding people’s interest in health topics to estimate cancer trends. Although most cancers examined in this study showed statistically significant correlations of the Baidu index with incidence and mortality rates, nasopharynx, uterine, stomach, esophageal, and testicular cancers did not show such correlations. This is probably attributable to various public health-related phenomena that may increase search volumes independent of disease metrics, such as the National Cancer Prevention Week held by the China Anti-Cancer Association (April of each year) or appearance of reports of cancer among public figures. After launch of a public health campaign for a disease, the information-search behavior associated with the disease will also increase [37]. For example, during the US annual breast cancer awareness campaign in October, online activity was stimulated and the number of Google searchers for “breast cancer” increased significantly [22,38]. For leukemia, uterine cancer, and Hodgkin lymphoma, the relative Baidu index was negatively correlated with cancer mortality rates. The possible reason for this might be the differences in the amount of data. The search index values for these three cancers gradually increased with time and showed large absolute values (Multimedia Appendix 2), and their mortality rates were low and stable over time. The trend of search engine search terms may be affected by other factors such as public panic [39]. Owing to the convenient use of the internet and the reports on internet media, people are more familiar with the three abovementioned cancers. Similarly, interest in breast cancer increased in January 2015 in China, perhaps because of the death of the well-known singer Beina Yao due to breast cancer.

In terms of the gender distribution of the search population, there were initially more men than women in our study. This could be attributable to gender differences in the disease burden pattern. For example, men are more susceptible than women to various deadly diseases, including cancer [40,41]. The gradual increase in the percentage of women searching for cancer-related information (especially for breast, ovarian, and uterine cancers) reflects increased health awareness among women. For other cancers, the gender structure of internet users tended to be balanced and basically consistent with the sex ratio of the population. In the process of obtaining search index data, we also found that people aged over 50 years were most prone to cancer among all age groups. The proportion of this age group among people who search online for cancer-related information is low, because they are less familiar with mobile devices and internet use [42]. Therefore, cancer-prevention initiatives should pay more attention to this age group to help them understand the relevant information. China is a vast and diverse country, with a population of more than 1.3 billion people. Regional differences were also found in search volumes, which could stem from regional disparities in demographic and socioeconomic conditions, education, and health literacy. For example, eastern China ranks first in search volumes, whereas less developed areas such as the northwest ranked last. People in densely populated and economically developed cities in eastern China had better internet access and higher health awareness. They search for health information more frequently than people in sparsely populated and developing cities. Local authorities should make efforts to ensure that online health information is accessible to the public, especially in economically underdeveloped areas.

Given the nonstandard treatments and other related issues, cancer diagnoses in China are generally made late, and the survival rates are not high [7]. Establishing effective cancer-control measures has thus become an important public health issue in China. Studies have shown that tracking and monitoring search index values as well as text mining on social media can provide new ways to study public concerns about cancer and information-seeking behaviors [12]. Web search content could provide valuable data for research on cancer-related topics [43,44].

Norman and Skinner defined eHealth literacy as “The ability to search, understand, and evaluate health information on electronic resources, and harnessing the information they receive to address and solve health problems”. As the content of health literacy continued to expand, Norman and Skinner proposed that electronic health literacy is a combination of different abilities, which can be divided into two types: traditional literacy vs computing ability, media literacy, and information literacy. Computer literacy, scientific literacy, and health literacy refer to the ability to deal specifically with problems in specific areas [45,46]. At present, there is little research in China on cancer-related electronic health literacy (or health literacy, in general) despite the fact that public retrieval of cancer-related health information indirectly reflects individual levels of electronic health literacy. It is necessary to enhance the efficiency of prevention and early diagnosis for patients with cancer or the general population by online information transmission. Collection of real-time relevant search data from search engines provides a new way for cancer prevention and control.

Research on attention paid to health information in the Web as well as population characteristics can help estimate some indicators of diseases among the population, which can help improve the allocation of health resources and implementation of effective public health measures. This could also help medical providers who are facing various challenges including understanding characteristics of patients who use the internet, the reasons for utilizing the internet, and the effectiveness and security of websites currently providing health-related information to patients.

### Strengths and Limitations

Previous studies have mainly used Google Trends [47] or the Baidu index to analyze the burden of epidemic diseases and predict their trends. This is the first study to explore the associations between online interest, cancer incidence, and mortality rates in China.

This study has some limitations. The use of Baidu search data to estimate disease metrics might not be completely generalizable, since the data are restricted to those with access to the internet. We were also unable to determine the types of internet users or which stakeholders were responsible for search activities. Given China’s vast size and large population, the registry of cancer statistics is usually lagging and not comprehensive. We could not obtain timely data on the monthly incidence and mortality rates of all cancers in China. Use of search index values from a popular internet search engine can only account for a small portion of changes in incidence and mortality rates of cancers, which are also greatly affected by public health activities. Studying search engine data is inevitably restricted by these random factors; this is an unavoidable limitation in such research. We hope to find ways to identify and reduce bias in search engine data before we utilize Web-based data to provide useful information for cancer surveillance, evaluation of public cancer awareness, and education programs.

### Conclusions

Owing to the widespread proliferation of internet technology, all kinds of people make use of the internet. In the medical field, it is often intended to prompt informed conversations with clinical professionals and suggest potentially helpful resources to patients or other people. Indeed, this study found a correlation between search index values and the incidence and mortality rates for most types of cancers. In a way, search behaviors and volumes can reflect the public awareness of cancer. Therefore, an advanced understanding of search behaviors could augment traditional epidemiologic surveillance and help achieve the goal of cancer prevention and control. It will be beneficial for us to pay attention to internet search data, especially when registry data are insufficient or lagging.

## Appendix

Multimedia Appendix 1 Search terms of 28 cancers in Chinese.

Multimedia Appendix 2 Relevant figures of the remaining types of cancers.

Multimedia Appendix 3 Results of unit root tests for the time series of monthly Baidu index, incidence rate, and mortality rate for each cancer type.

Multimedia Appendix 4 Results of the cointegration test of the two-time series of monthly Baidu indexes and incidence rates of cancers.

Multimedia Appendix 5 Results of the cointegration test of the two-time series of monthly Baidu indexes and mortality rates of cancers.
