# Supplementary material for: Association Between Cancer Incidence and Mortality in Web-Based Data in China: Infodemiology Study
Source: J Med Internet Res. 2019 Jan 29;21(1):e10677. doi: 10.2196/10677 (PMC6371071; doi:10.2196/10677)
Supplement: Multimedia Appendix 1 [file jmir_v21i1e10677_app1.pdf]

Table 1. Search terms of 28 cancers in Chinese.

Cancer type	Key words of each cancer	Numbers of the key words
Lung cancer	肺癌+肺腺癌+肺鳞癌+肺部肿瘤	4
Liver cancer	肝癌+肝肿瘤+肝脏肿瘤	3
Stomach cancer	胃癌+胃腺癌	2
Esophageal cancer	食管癌+食道癌	2
Colon and rectal cancer	结肠癌+大肠癌+肠癌	6
	直肠癌+大肠癌+肠癌	
Pancreatic cancer	胰腺癌+胰头癌+消化道肿瘤+胰腺肿瘤	4
Breast cancer	乳腺癌+乳癌+乳腺肿瘤	3
Leukemia	白血病+血癌	2
Brain and nervous system cancer	脑瘤+脑癌+颅内肿瘤+脑部肿瘤	4
Cervical cancer	宫颈癌+子宫颈癌	2
Non-Hodgkin lymphoma	非霍奇金淋巴瘤+淋巴瘤+淋巴肿瘤	3
Prostate cancer	前列腺癌	1
Nasopharyngeal cancer	鼻咽癌+鼻癌+咽喉癌	3
Bladder cancer	膀胱癌+膀胱肿瘤	2
Gallbladder and biliary tract cancer	胆囊癌+胆管癌+胆管肿瘤	3
Lip and cavity cancer	口腔癌+口底癌+牙龈癌+舌癌	4
Ovarian cancer	卵巢癌+卵巢肿瘤+卵巢恶性肿瘤	3
Larynx cancer	喉癌	1
Kidney cancer	肾癌+肾细胞癌+肾肿瘤+肾脏肿瘤	4
Squamous-cell carcinoma	鳞状细胞癌+鳞癌	2
Uterine cancer	子宫癌+子宫内膜癌	2
Multiple myeloma	多发性骨髓瘤+骨髓瘤	2
Thyroid cancer	甲状腺癌+甲状腺肿瘤	2
Malignant skin melanoma	恶性黑色素瘤+恶性黑素瘤+黑色素瘤	3
Hodgkin lymphoma	霍奇金淋巴瘤	1
Mesothelioma	间皮瘤	1
Testicular cancer	睾丸癌+睾丸肿瘤	2
Basal-cell carcinoma	基底细胞癌+基底细胞瘤	2
