# Supplementary material for: Association Between Cancer Incidence and Mortality in Web-Based Data in China: Infodemiology Study
Source: J Med Internet Res. 2019 Jan 29;21(1):e10677. doi: 10.2196/10677 (PMC6371071; doi:10.2196/10677)
Supplement: Multimedia Appendix 2 [file jmir_v21i1e10677_app2.pdf]

Figure 1. Time series of search index values, incidence and mortality rates of the reaming cancers. Monthly search index values of cancers from 2011-2016 were obtained from www.index.baidu.com. Incidence and mortality rates of cancers from 2011-2016 were obtained from Global Burden of Disease Database (GBD).

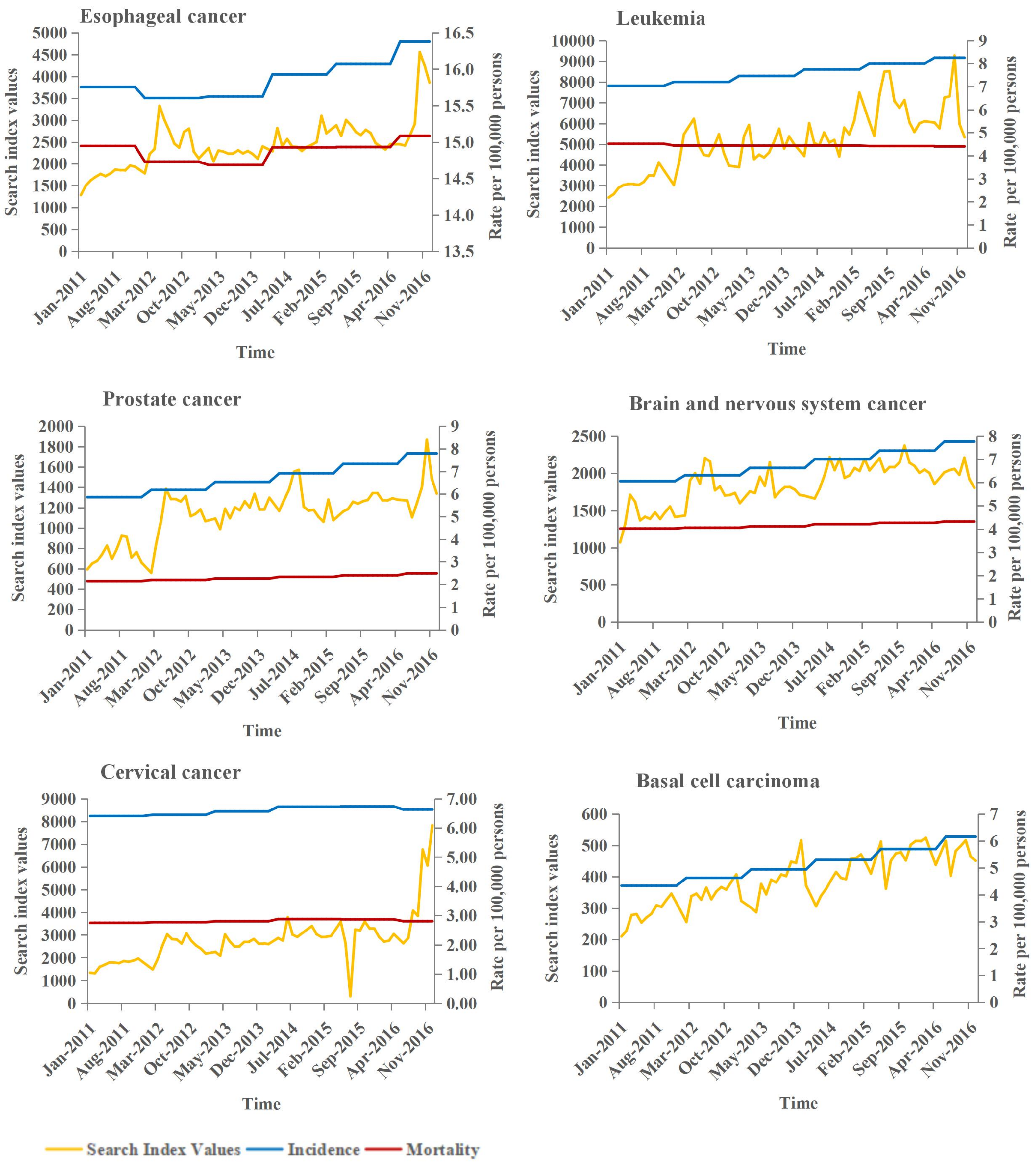

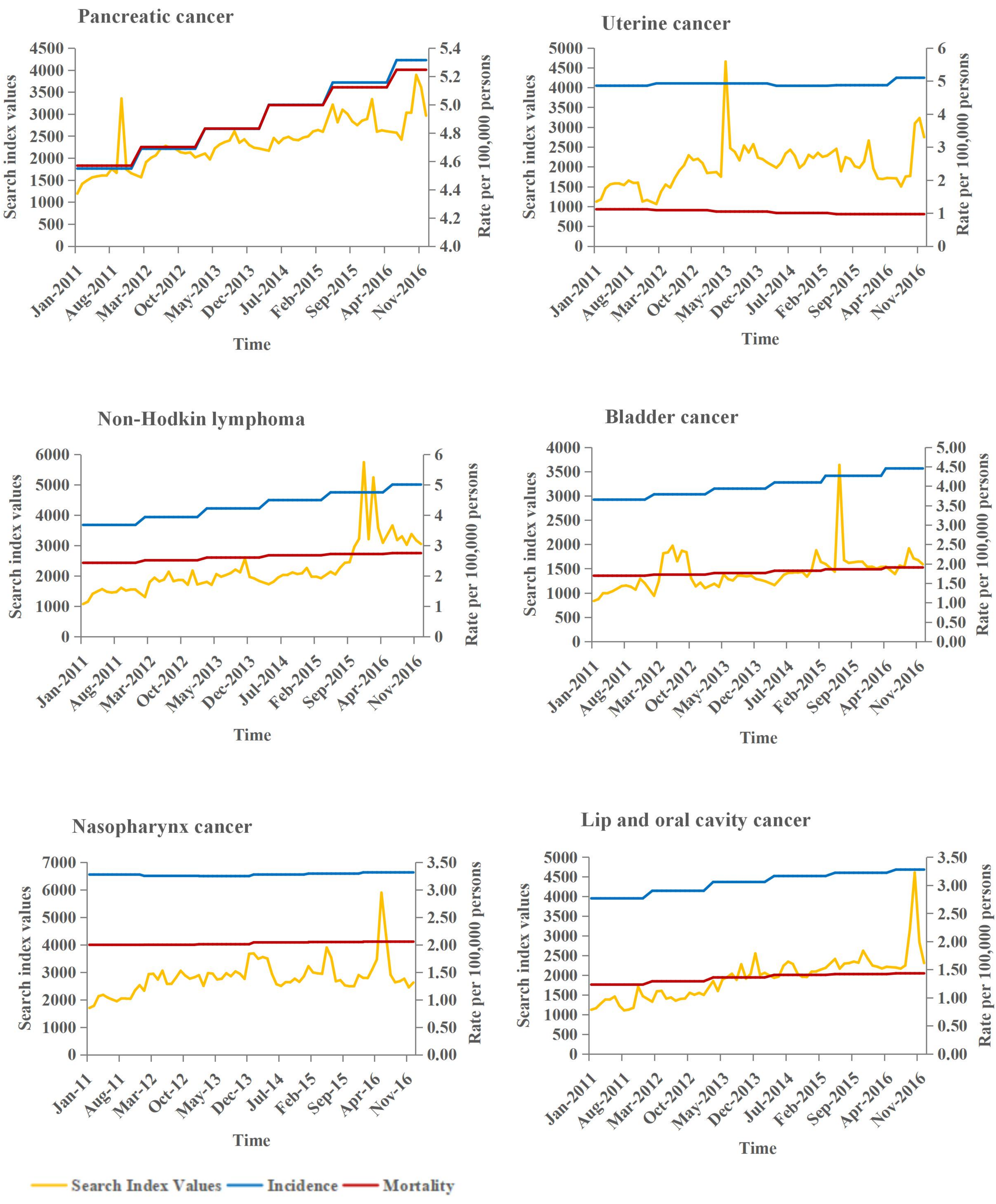
Pancreatic cancer
Uterine cancer
Non-Hodkin lymphoma
Bladder cancer
Nasopharynx cancer
Lip and oral cavity cancer
Search index values
Rate per 100,000 persons
Time
Jan-2011
Aug-2011
Mar-2012
Oct-2012
May-2013
Dec-2013
Jul-2014
Feb-2015
Sep-2015
Apr-2016
Nov-2016
Jan-11
Aug-11
Mar-12
Oct-12
May-13
Dec-13
Jul-14
Feb-15
Sep-15
Apr-16
Nov-16
Search Index Values
Incidence
Mortality

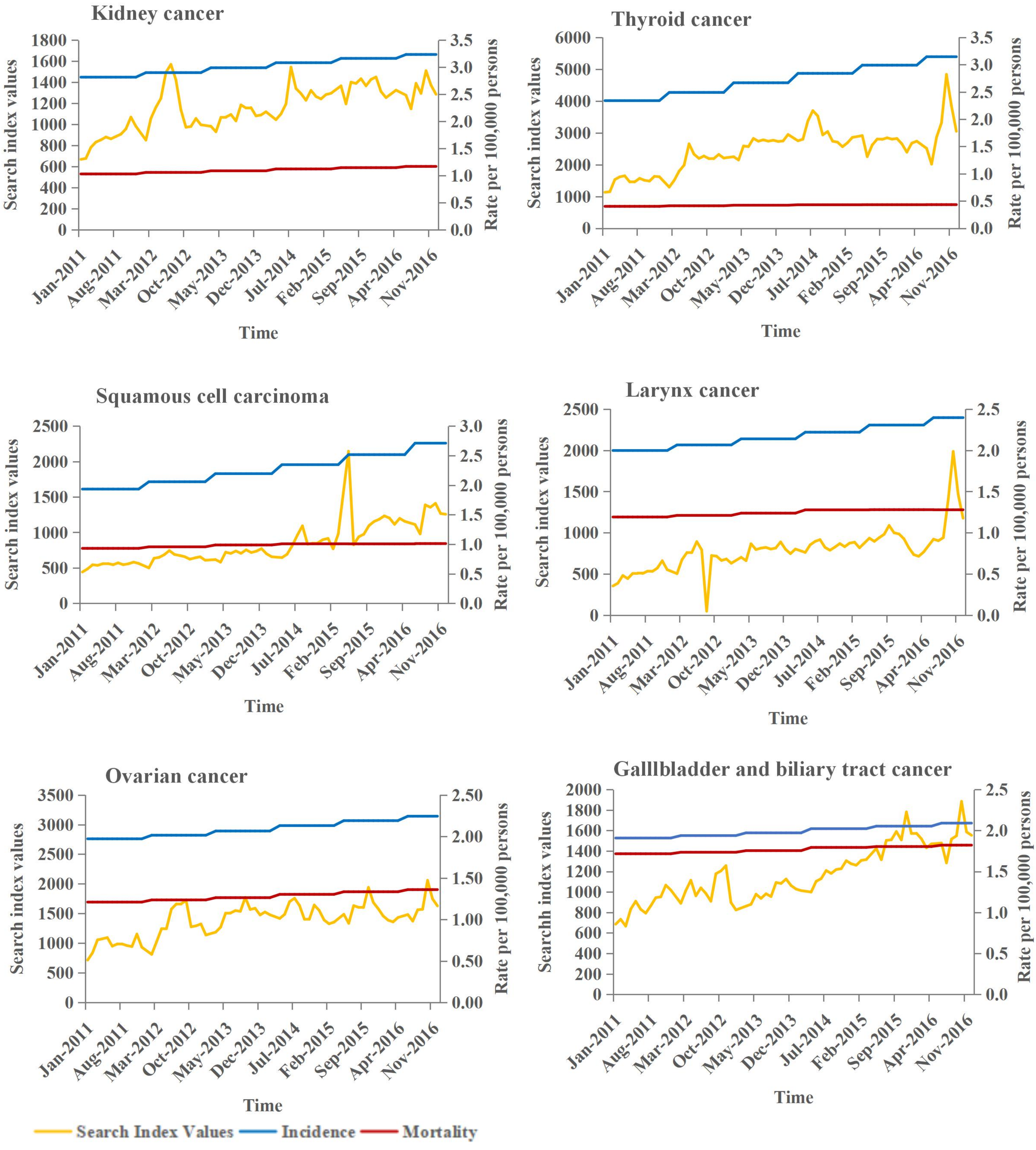
Kidney cancer
Thyroid cancer
Squamous cell carcinoma
Larynx cancer
Ovarian cancer
Galllbladder and biliary tract cancer
Search index values
Searchh index values
Rate per 100,000 persons
Time
Jan-2011
Aug-2011
Mar-2012
Oct-2012
May-2013
Dec-2013
Jul-2014
Feb-2015
Sep-2015
Apr-2016
Nov-2016
Search Index Values
Incidence
Mortality

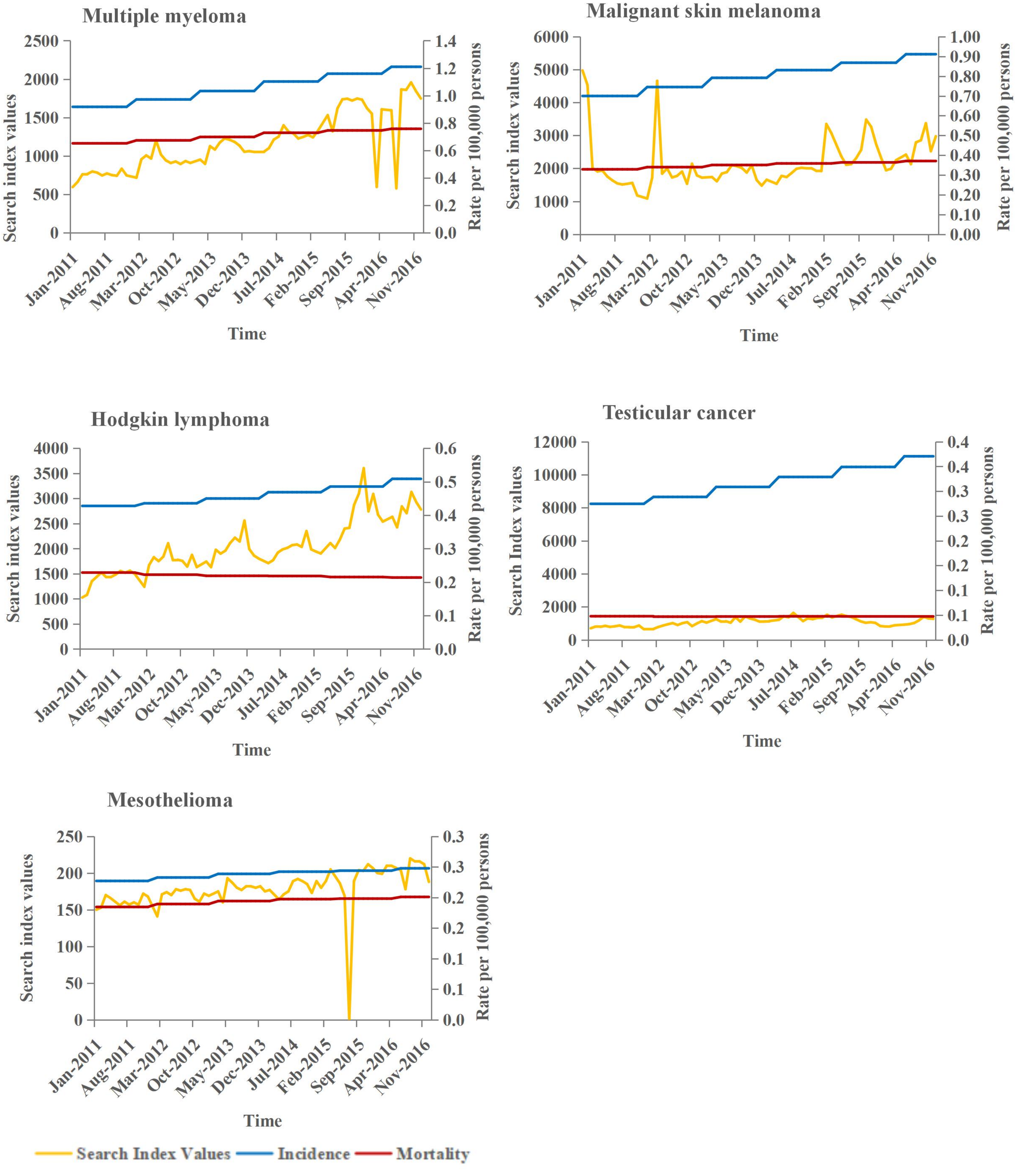
Multiple myeloma
Malignant skin melanoma
Hodgkin lymphoma
Testicular cancer
Mesothelioma
Search index values
Search Index values
Rate per 100,000 persons
Time
Jan-2011
Aug-2011
Mar-2012
Oct-2012
May-2013
Dec-2013
Jul-2014
Feb-2015
Sep-2015
Apr-2016
Nov-2016
Search Index Values
Incidence
Mortality

Figure 2. Incidence, mortality, search sex distribution of each cancer divided by gender of the remaining cancers. The percentile chart represents the change in the sex distribution of the search population from September 2013 to September 2016. The blue line represents incidence rate of each cancer. The red line represents mortality rate of each cancer.

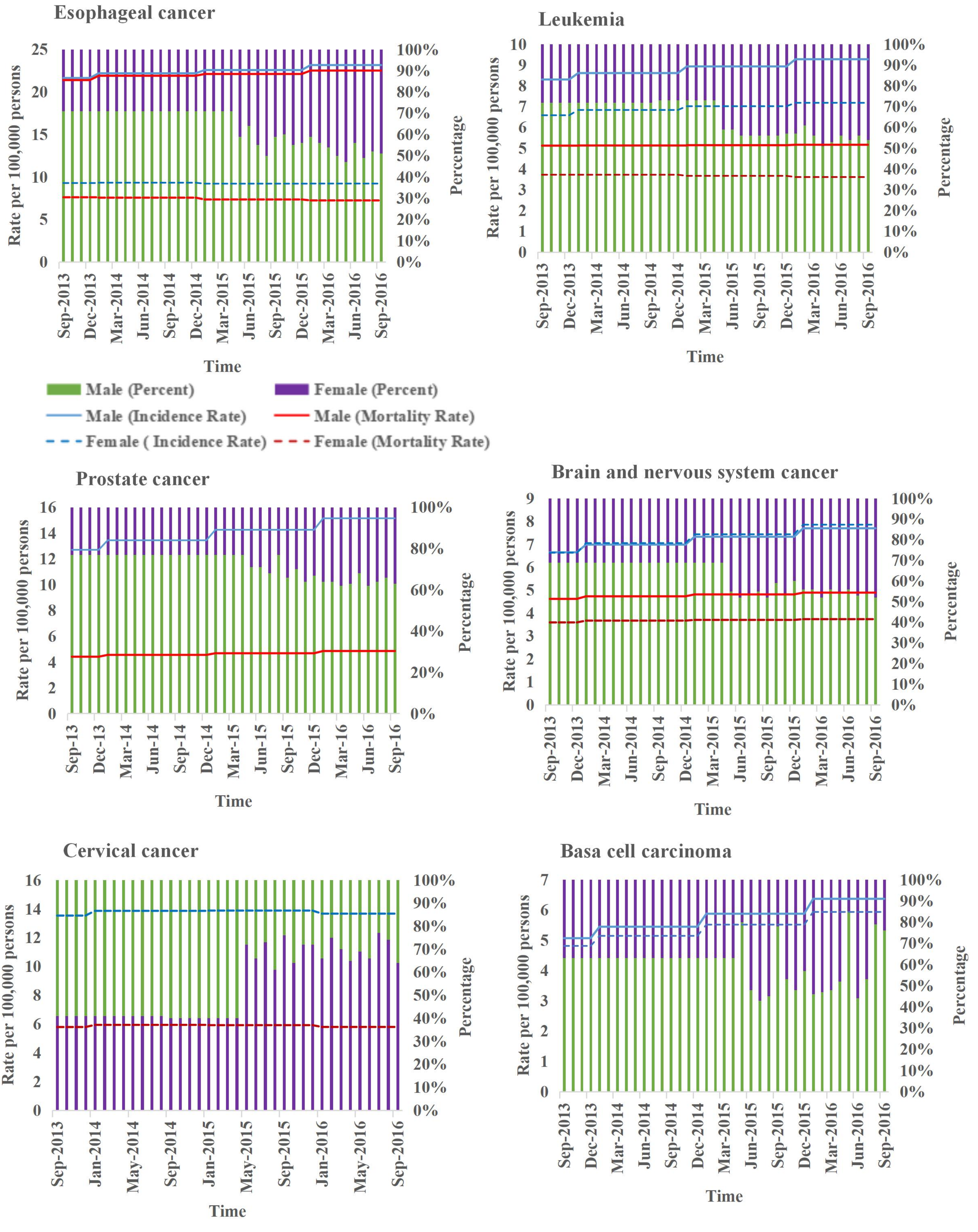

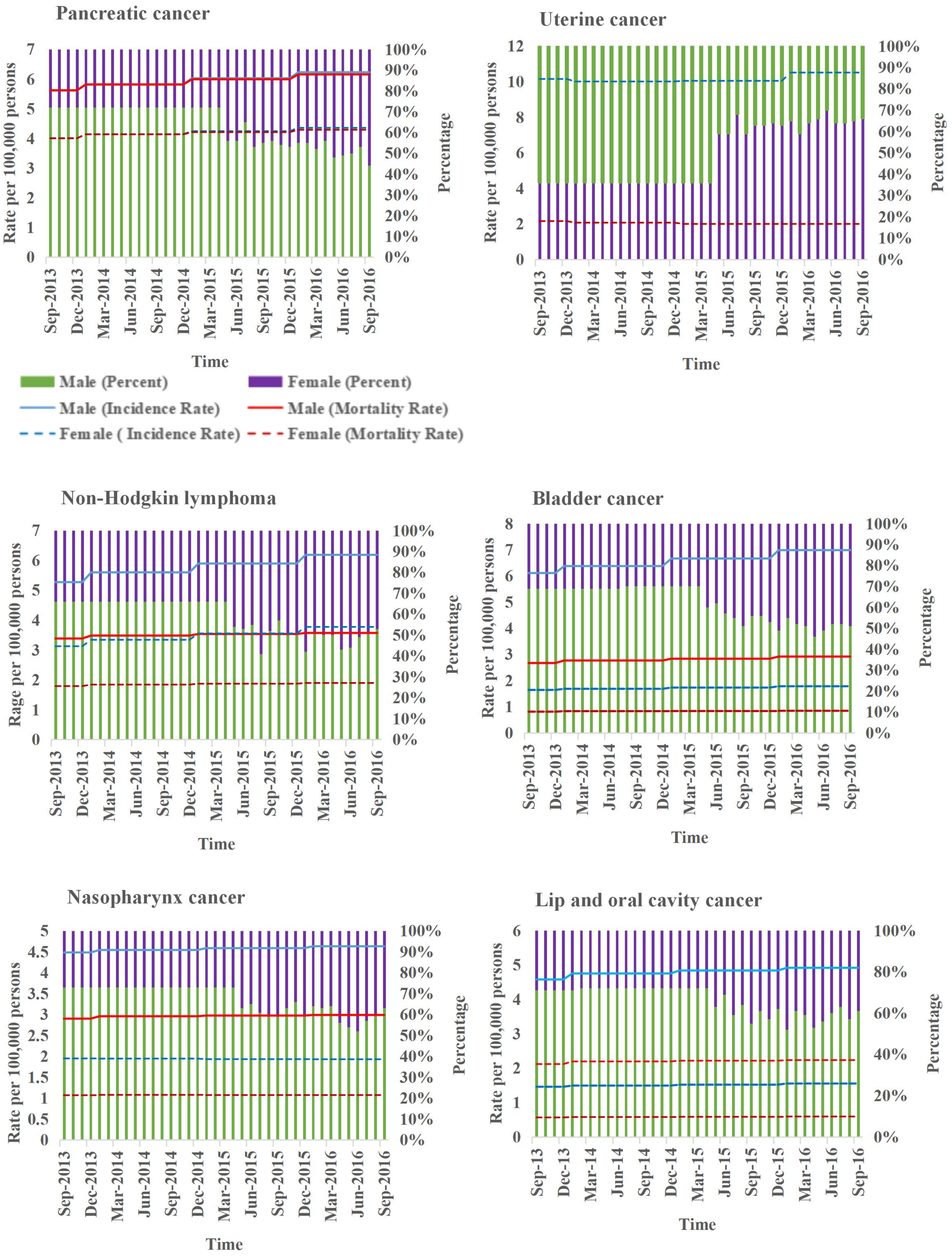
Pancreatic cancer
Uterine cancer
Non-Hodgkin lymphoma
Bladder cancer
Nasopharynx cancer
Lip and oral cavity cancer
Rate per 100,000 persons
Rage per 100,000 persons
Percentage
Time
Male (Percent)
Female (Percent)
Male (Incidence Rate)
Male (Mortality Rate)
Female (Incidence Rate)
Female (Mortality Rate)

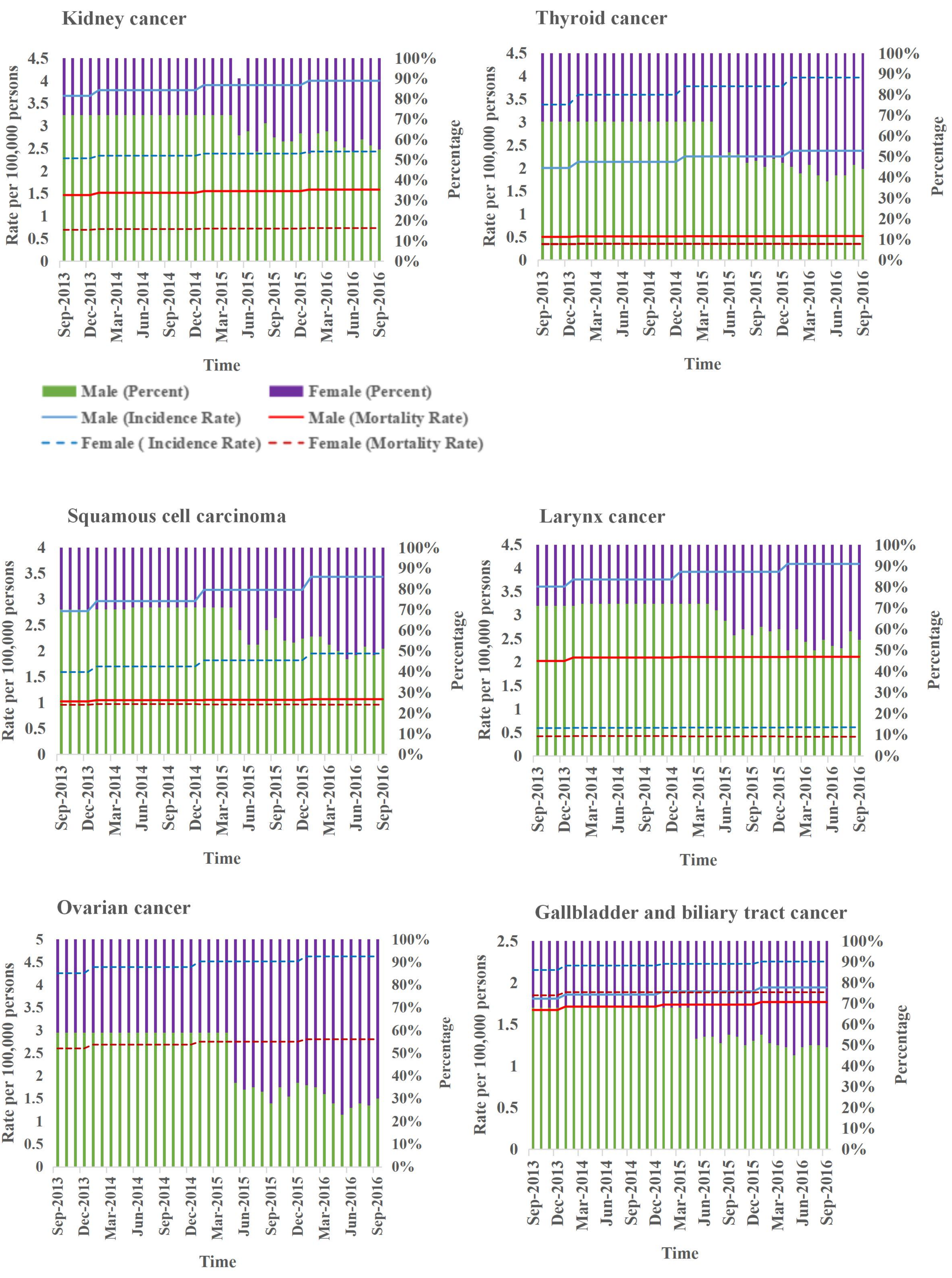
Kidney cancer
Thyroid cancer
Squamous cell carcinoma
Larynx cancer
Ovarian cancer
Gallbladder and biliary tract cancer
Rate per 100,000 persons
Percentage
Time
Sep-2013
Dec-2013
Mar-2014
Jun-2014
Sep-2014
Dec-2014
Mar-2015
Jun-2015
Sep-2015
Dec-2015
Mar-2016
Jun-2016
Sep-2016
Male (Percent)
Female (Percent)
Male (Incidence Rate)
Male (Mortality Rate)
Female (Incidence Rate)
Female (Mortality Rate)

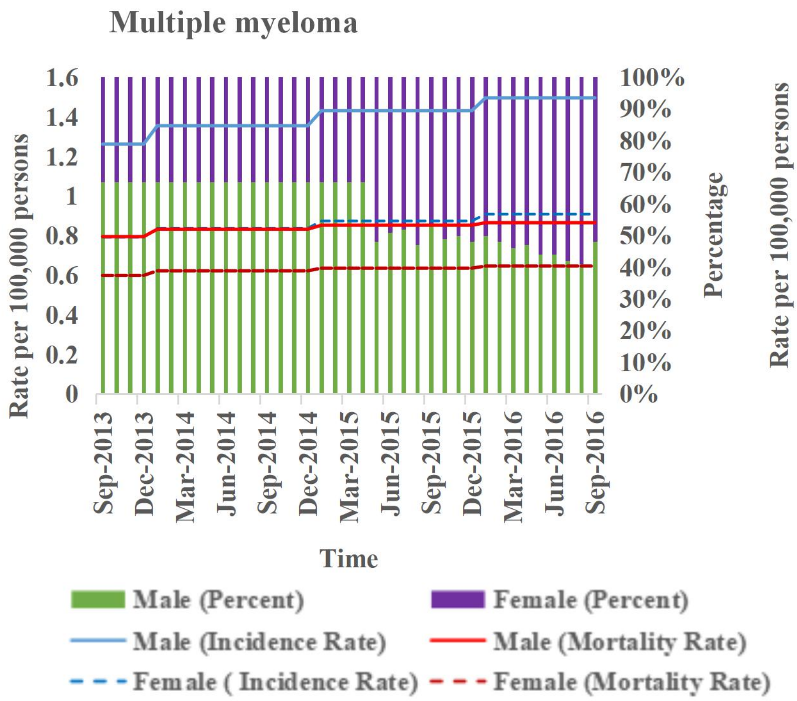
Multiple myeloma
Rate per 100,000 persons
Percentage
Time
Sep-2013
Dec-2013
Mar-2014
Jun-2014
Sep-2014
Dec-2014
Mar-2015
Jun-2015
Sep-2015
Dec-2015
Mar-2016
Jun-2016
Sep-2016
Male (Percent)
Female (Percent)
Male (Incidence Rate)
Male (Mortality Rate)
Female (Incidence Rate)
Female (Mortality Rate)

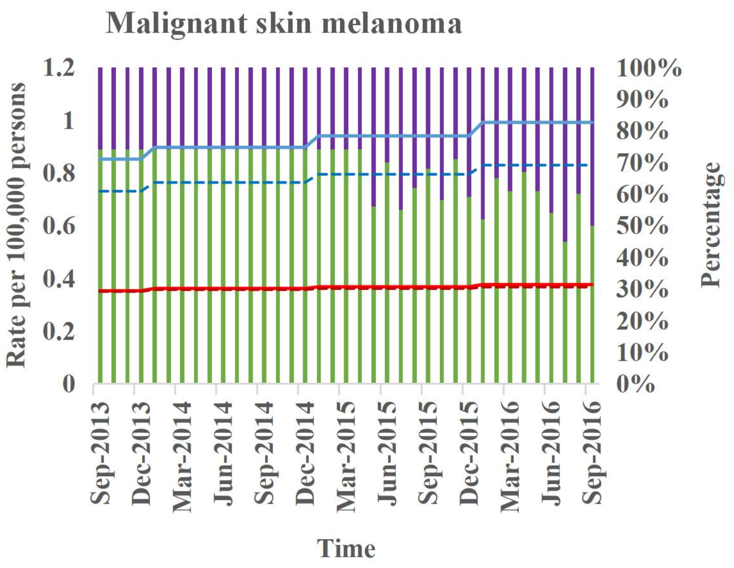
Malignant skin melanoma
Rate per 100,000 persons
Percentage
Time

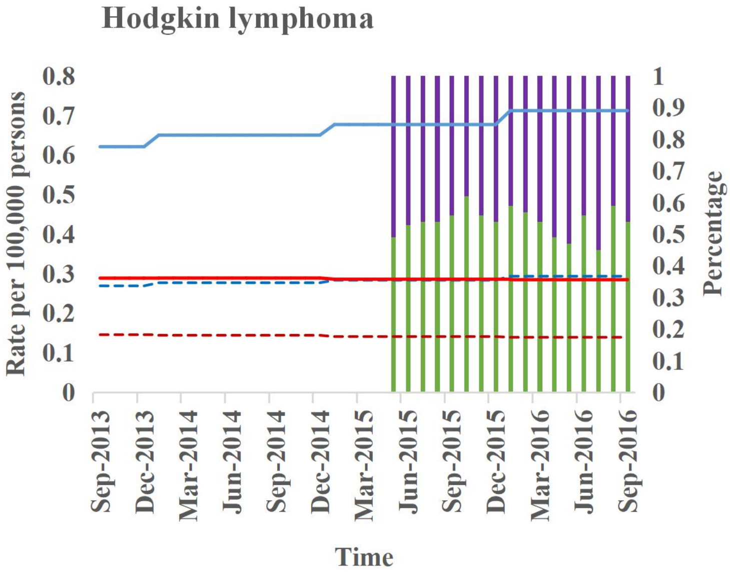
Hodgkin lymphoma
Rate per 100,000 persons
Percentage
Time

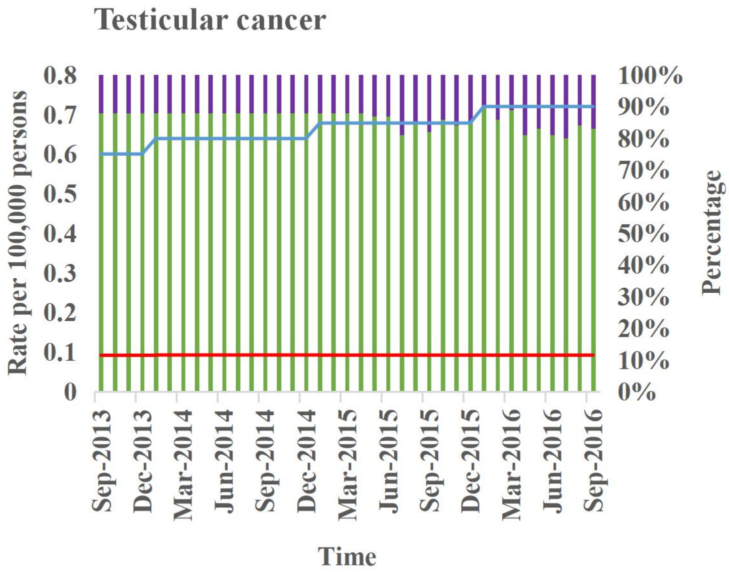
Testicular cancer
Rate per 100,000 persons
Percentage
Time

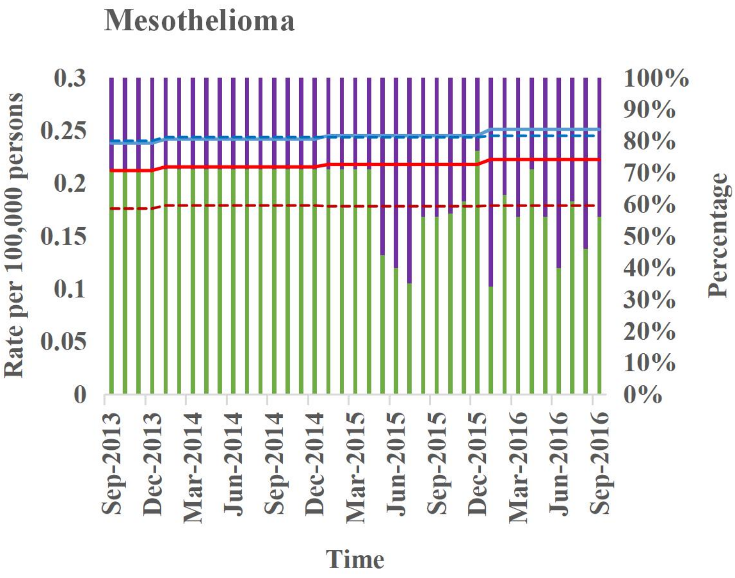
Mesothelioma
Rate per 100,000 persons
Percentage
Time

Figure 3. Age distribution of the online searchers from 2013 to 2016 (remaining cancers). Blue represents the age distribution of search people in 2013. Orange represents the situation in 2014. Gray represents the situation in 2015. Yellow represents the situation in 2016. Except for the top ten cancers, the remaining cancers, due to the lack of data, we only obtained the overall age distribution of the search population in these three years.

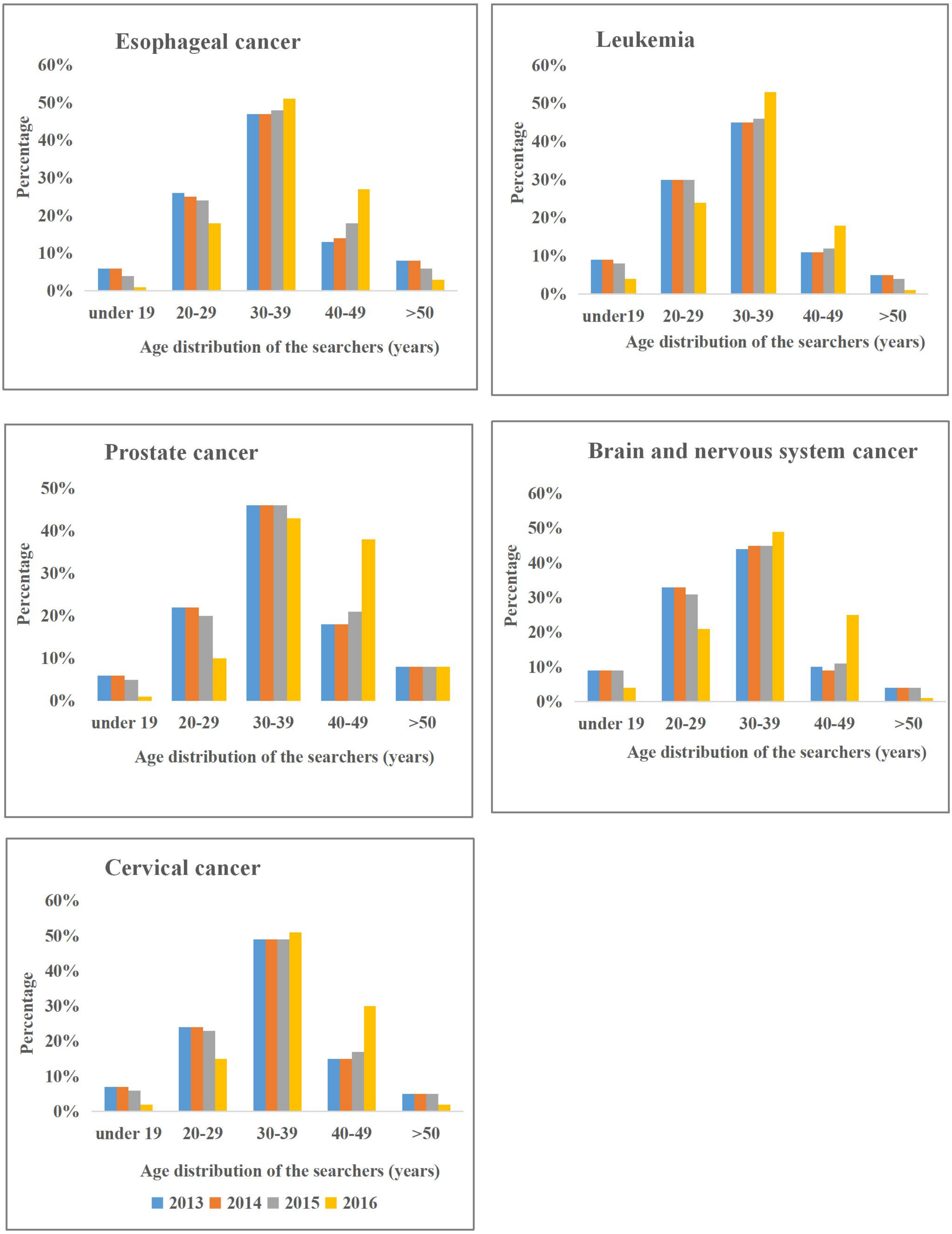

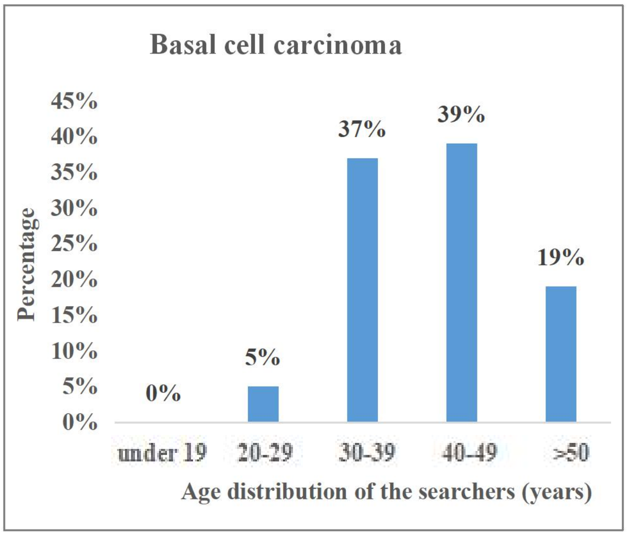
Basal cell carcinoma
Percentage
45%
40%
35%
30%
25%
20%
15%
10%
5%
0%
0%
5%
37%
39%
19%
under 19
20-29
30-39
40-49
>50
Age distribution of the searchers (years)

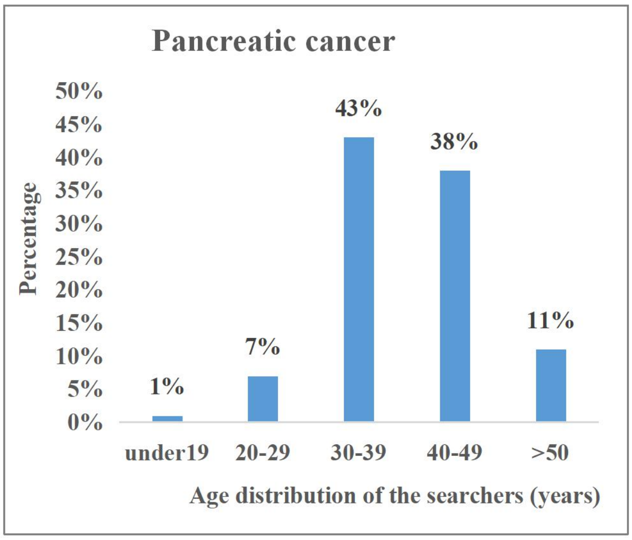
Pancreatic cancer
Percentage
50%
45%
40%
35%
30%
25%
20%
15%
10%
5%
0%
1%
7%
43%
38%
11%
under19
20-29
30-39
40-49
>50
Age distribution of the searchers (years)

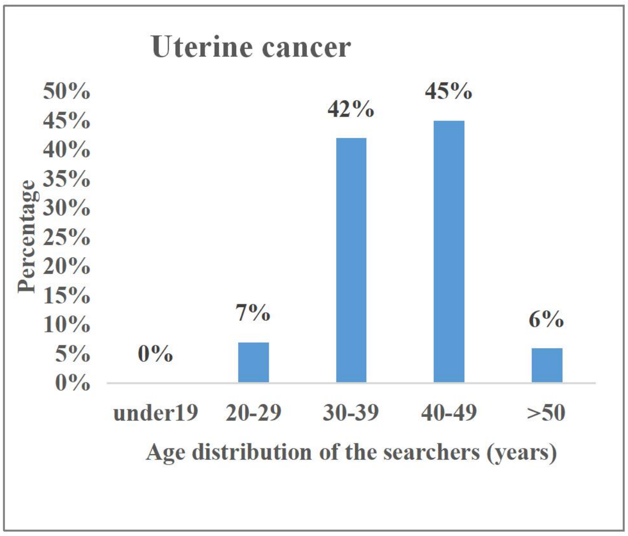
Uterine cancer
Percentage
50%
45%
40%
35%
30%
25%
20%
15%
10%
5%
0%
0%
7%
42%
45%
6%
under19
20-29
30-39
40-49
>50
Age distribution of the searchers (years)

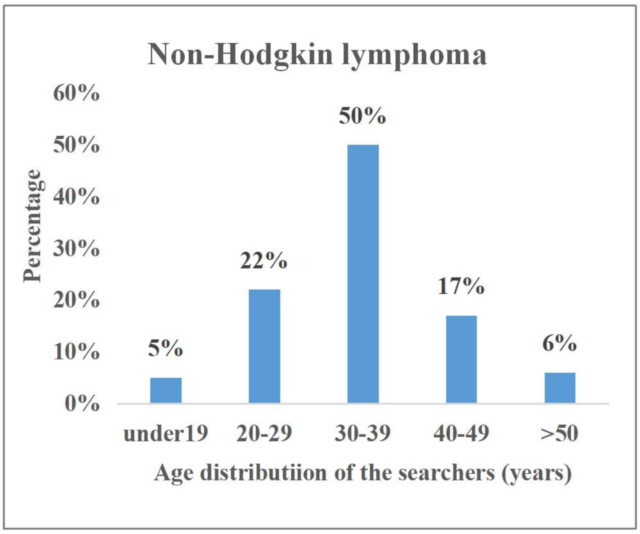
Non-Hodgkin lymphoma
Percentage
60%
50%
40%
30%
20%
10%
0%
5%
22%
50%
17%
6%
under19
20-29
30-39
40-49
>50
Age distributiion of the searchers (years)

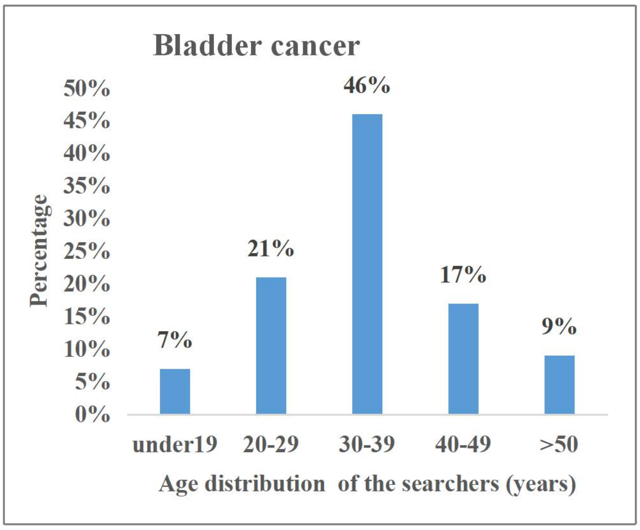
Bladder cancer
Percentage
50%
45%
40%
35%
30%
25%
20%
15%
10%
5%
0%
7%
21%
46%
17%
9%
under19
20-29
30-39
40-49
>50
Age distribution of the searchers (years)

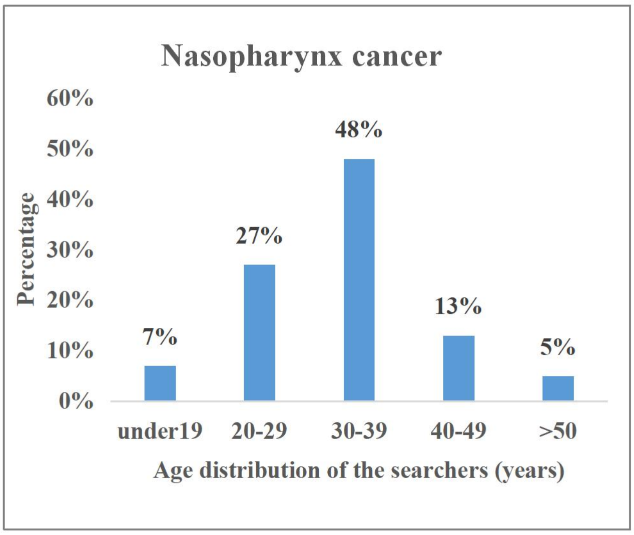
Nasopharynx cancer
Percentage
60%
50%
40%
30%
20%
10%
0%
7%
27%
48%
13%
5%
under19
20-29
30-39
40-49
>50
Age distribution of the searchers (years)

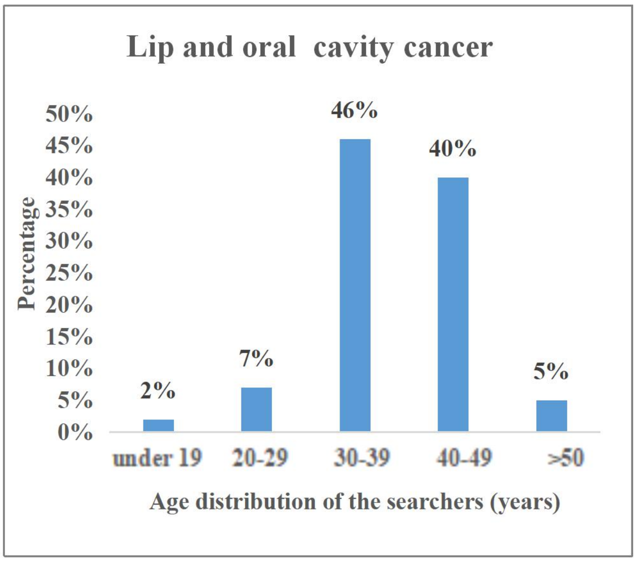
Lip and oral cavity cancer
Percentage
50%
45%
40%
35%
30%
25%
20%
15%
10%
5%
0%
2%
7%
46%
40%
5%
under 19
20-29
30-39
40-49
>50
Age distribution of the searchers (years)

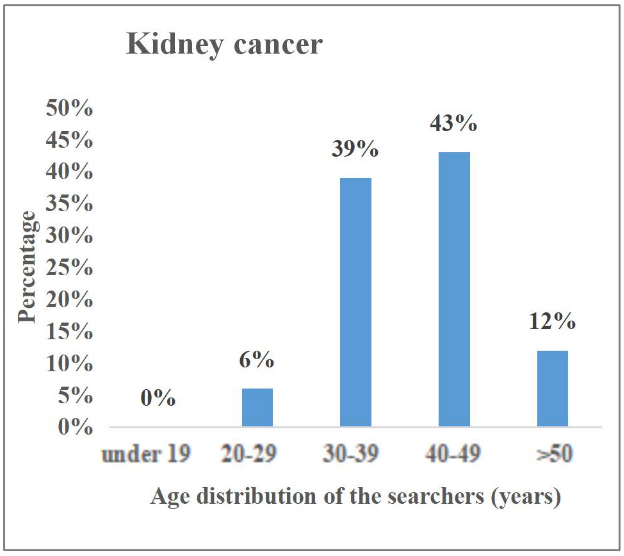
Kidney cancer
Percentage
50%
45%
40%
35%
30%
25%
20%
15%
10%
5%
0%
0%
6%
39%
43%
12%
under 19
20-29
30-39
40-49
>50
Age distribution of the searchers (years)

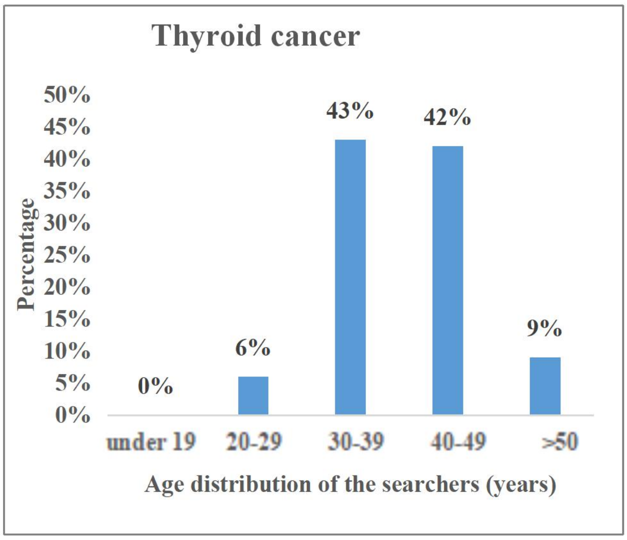
Thyroid cancer
Percentage
50%
45%
40%
35%
30%
25%
20%
15%
10%
5%
0%
0%
6%
43%
42%
9%
under 19
20-29
30-39
40-49
>50
Age distribution of the searchers (years)

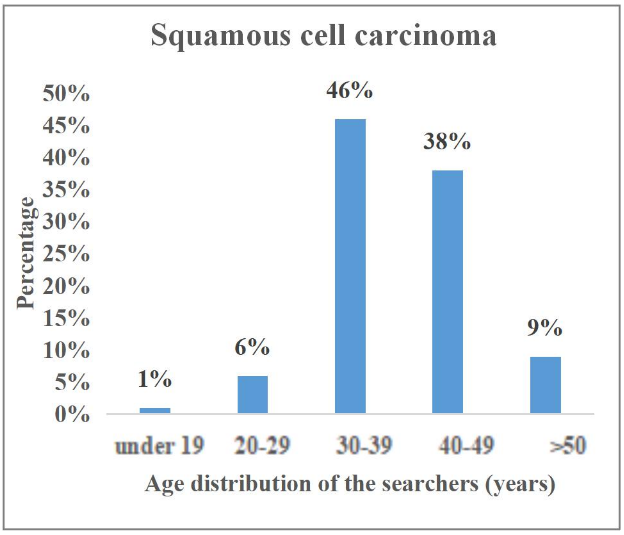
Squamous cell carcinoma
Percentage
50%
45%
40%
35%
30%
25%
20%
15%
10%
5%
0%
1%
6%
46%
38%
9%
under 19
20-29
30-39
40-49
>50
Age distribution of the searchers (years)

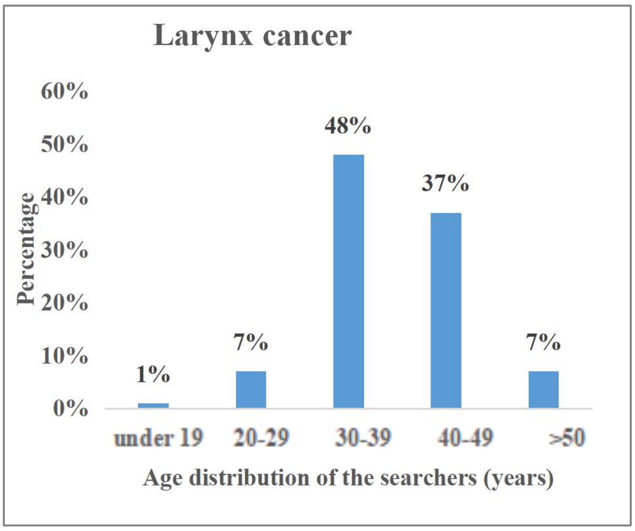
Larynx cancer
Percentage
60%
50%
40%
30%
20%
10%
0%
1%
7%
48%
37%
7%
under 19
20-29
30-39
40-49
>50
Age distribution of the searchers (years)

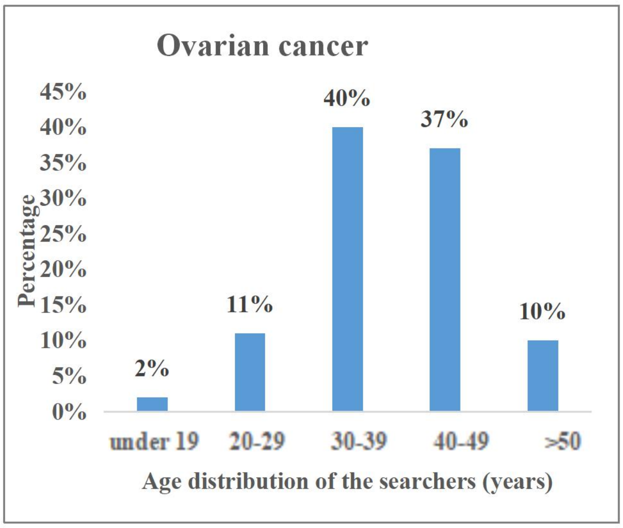
Ovarian cancer
Percentage
45%
40%
35%
30%
25%
20%
15%
10%
5%
0%
2%
11%
40%
37%
10%
under 19
20-29
30-39
40-49
>50
Age distribution of the searchers (years)

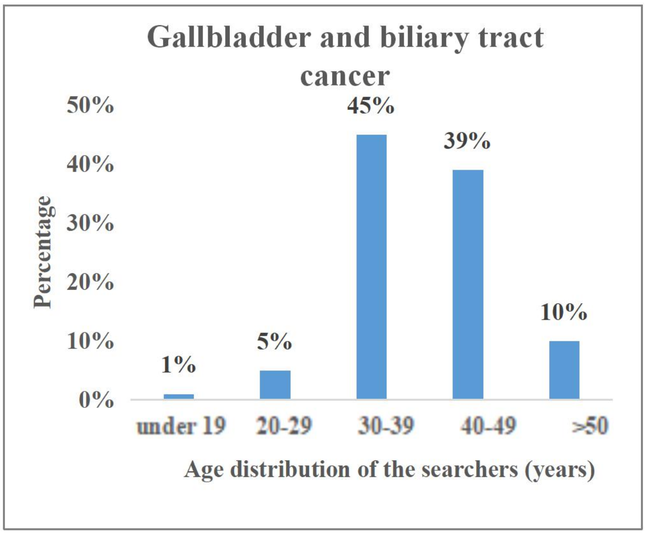
Gallbladder and biliary tract cancer
Percentage
50%
40%
30%
20%
10%
0%
1%
5%
45%
39%
10%
under 19
20-29
30-39
40-49
>50
Age distribution of the searchers (years)

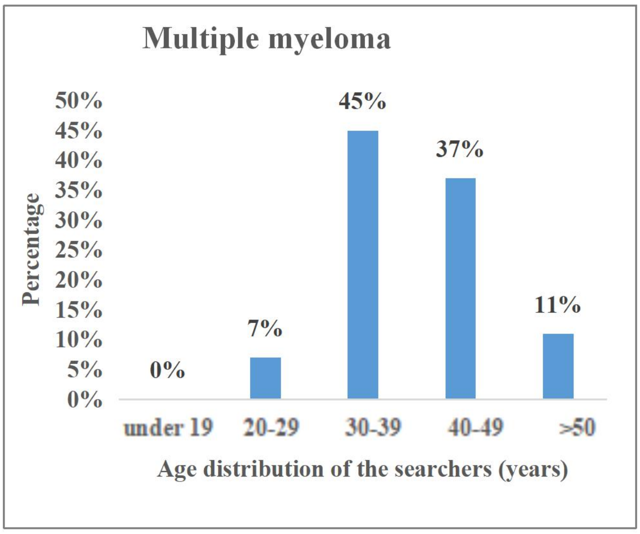
Multiple myeloma
Percentage
50%
45%
40%
35%
30%
25%
20%
15%
10%
5%
0%
0%
7%
45%
37%
11%
under 19
20-29
30-39
40-49
>50
Age distribution of the searchers (years)

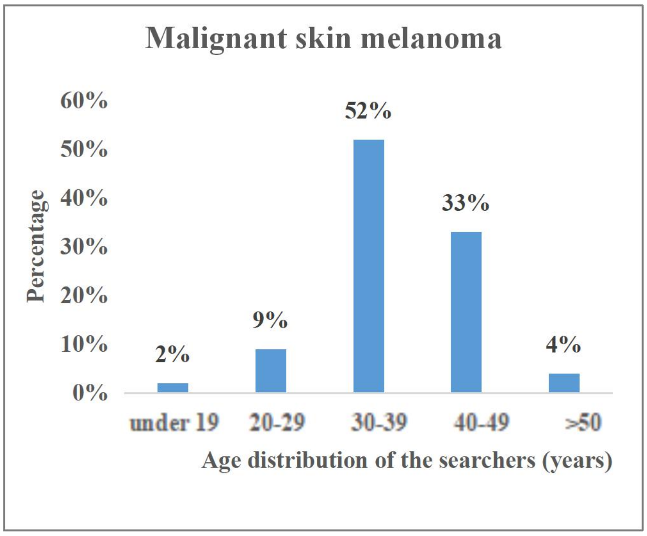
Malignant skin melanoma
Percentage
60%
50%
40%
30%
20%
10%
0%
2%
9%
52%
33%
4%
under 19
20-29
30-39
40-49
>50
Age distribution of the searchers (years)

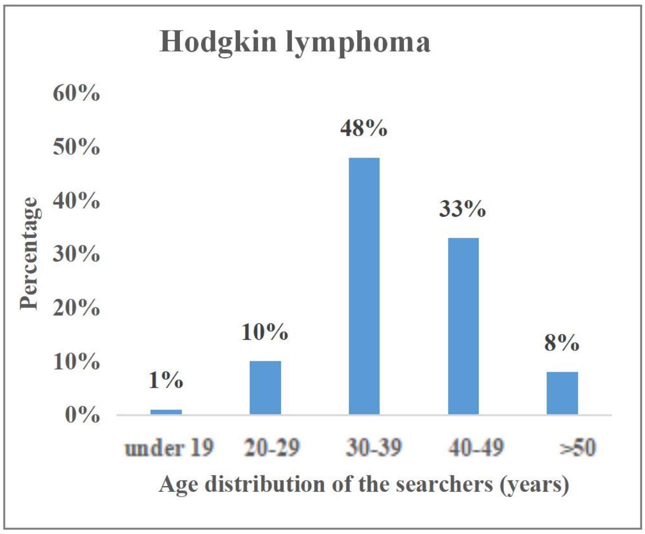
Hodgkin lymphoma
Percentage
60%
50%
40%
30%
20%
10%
0%
1%
10%
48%
33%
8%
under 19
20-29
30-39
40-49
>50
Age distribution of the searchers (years)

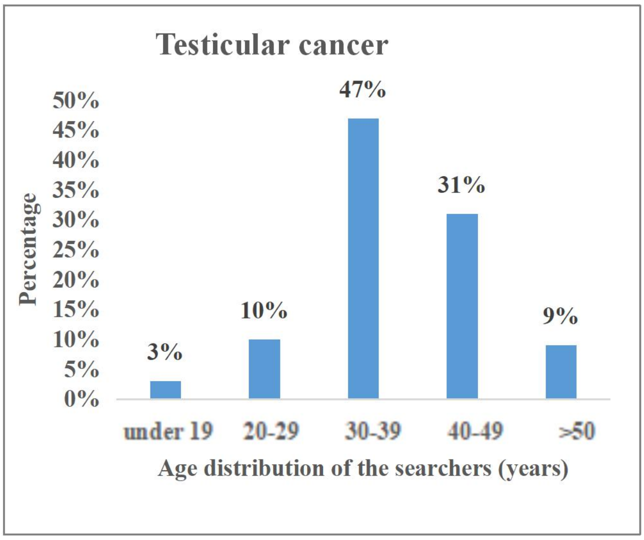
Testicular cancer
Percentage
50%
45%
40%
35%
30%
25%
20%
15%
10%
5%
0%
3%
10%
47%
31%
9%
under 19
20-29
30-39
40-49
>50
Age distribution of the searchers (years)

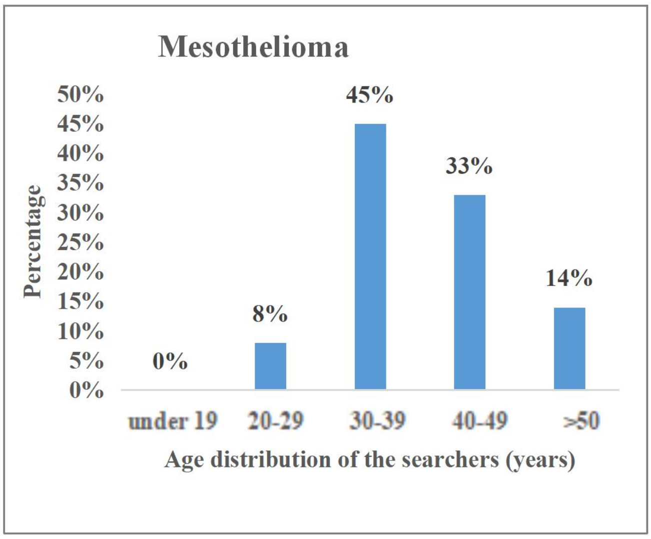
Mesothelioma
Percentage
50%
45%
40%
35%
30%
25%
20%
15%
10%
5%
0%
0%
8%
45%
33%
14%
under 19
20-29
30-39
40-49
>50
Age distribution of the searchers (years)
