# Supplementary material for: Association Between Cancer Incidence and Mortality in Web-Based Data in China: Infodemiology Study
Source: J Med Internet Res. 2019 Jan 29;21(1):e10677. doi: 10.2196/10677 (PMC6371071; doi:10.2196/10677)
Supplement: Multimedia Appendix 3 [file jmir_v21i1e10677_app3.pdf]

Table 2. Abbreviations and full names of the 31 provinces and cities

Number	Abbreviation	Full Name
1	AH	Anhui
2	BJ	Beijing
3	FJ	Fujian
4	GS	Gansu
5	GD	Guangdong
6	GX	Guangxi
7	GZ	Guizhou
8	HI	Hainan
9	HE	Hebei
10	HA	Henan
11	HL	Heilongjiang
12	HB	Hubei
13	HN	Hunan
14	JL	Jilin
15	JS	Jiangsu
16	JX	Jiangxi
17	LN	Liaoning
18	NM	Inner Mongoria
19	NX	Ningxia
20	QH	Qinghai
21	SD	Shandong
22	SX	Shanxi
23	SN	Shaanxi
24	SH	Shanghai
25	SC	Sichuan
26	TJ	Tianjing
27	XZ	Tibet
28	XJ	Xinjiang
29	YN	Yunnan
30	ZJ	Zhejiang
31	CQ	Chongqing
