# Supplementary material for: Association Between Cancer Incidence and Mortality in Web-Based Data in China: Infodemiology Study
Source: J Med Internet Res. 2019 Jan 29;21(1):e10677. doi: 10.2196/10677 (PMC6371071; doi:10.2196/10677)
Supplement: Multimedia Appendix 4 [file jmir_v21i1e10677_app4.pdf]

Table 3. Results of cointegration test of the two-time series of monthly Baidu indexes and incidence rates of cancers.

Cancer type	Unit root test for the residual series								Result [c]
	ADF	1% Level	5% Level	10% Level	*P* value	R^2	AIC [a] value	SC [b] value	
Lung cancer	-9.25	-4.09	-3.48	-3.17	<.001	0.56	-1.87	-1.78	Co-integration
Liver cancer	-8.66	-4.09	-3.48	-3.17	<.001	0.53	-5.16	-5.07	Co-integration
Stomach cancer	-8.58	-4.09	-3.48	-3.17	<.001	0.52	-5.30	-5.20	Co-integration
Colon and rectal cancer	-10.76	-4.09	-3.48	-3.17	<.001	0.63	-3.91	-3.82	Co-integration
Breast cancer	-12.38	-4.09	-3.48	-3.17	<.001	0.70	-4.42	-4.33	Co-integration
Esophageal cancer	-9.51	-4.09	-3.48	-3.17	<.001	0.57	-7.43	-7.33	Co-integration
Leukemia	-9.48	-4.09	-3.48	-3.17	<.001	0.57	-4.41	-4.32	Co-integration
Prostate cancer	-8.64	-4.09	-3.48	-3.17	<.001	0.53	-3.60	-3.50	Co-integration
Brain and nervous system cancer	-9.66	-4.09	-3.48	-3.17	<.001	0.61	-1.96	-1.86	Co-integration
Cervical cancer	-11.04	-4.09	-3.48	-3.17	<.001	0.65	-6.76	-6.66	Co-integration
Basal-cell carcinoma	-9.28	-4.09	-3.48	-3.17	<.001	0.56	-2.82	-2.72	Co-integration
Pancreatic cancer	-12.51	-4.09	-3.48	-3.17	<.001	0.70	-4.42	-4.33	Co-integration
Uterine cancer	-8.64	-4.09	-3.48	-3.17	<.001	0.53	-7.18	-7.08	Co-integration
Non-Hodgkin lymphoma	-13.18	-4.09	-3.48	-3.17	<.001	0.72	-3.41	-3.31	Co-integration
Bladder cancer	-11.21	-4.09	-3.48	-3.17	<.001	0.65	-3.97	-3.87	Co-integration
Nasopharynx cancer	-7.34	-4.09	-3.48	-3.17	<.001	0.46	-0.97	-0.87	Co-integration
Lip and oral cavity cancer	-9.02	-4.09	-3.48	-3.17	<.001	0.55	-4.20	-4.10	Co-integration
Kidney cancer	-8.46	-4.09	-3.48	-3.17	<.001	0.52	-4.95	-4.86	Co-integration
Thyroid cancer	-8.04	-4.09	-3.48	-3.17	<.001	0.49	-3.52	-3.42	Co-integration
Squamous-cell carcinoma	-10.99	-4.09	-3.48	-3.17	<.001	0.64	-2.97	-2.87	Co-integration
Larynx cancer	-13.02	-4.09	-3.48	-3.17	<.001	0.72	-3.57	-3.47	Co-integration
Ovarian cancer	-9.43	-4.09	-3.48	-3.17	<.001	0.57	-4.65	-4.55	Co-integration
Gallbladder and biliary tract cancer	-9.83	-4.09	-3.48	-3.17	<.001	0.59	-5.90	-5.81	Co-integration
Multiple myeloma	-13.18	-4.09	-3.48	-3.17	<.001	0.72	-2.80	-2.70	Co-integration
Malignant skin melanoma	-9.68	-4.09	-3.48	-3.17	<.001	0.58	-4.10	-4.00	Co-integration
Hodgkin lymphoma	-10.48	-4.09	-3.48	-3.17	<.001	0.62	-4.60	-4.50	Co-integration
Testicular cancer	-13.90	-4.09	-3.48	-3.17	<.001	0.74	-3.26	-3.17	Co-integration
Mesothelioma	-10.42	-4.09	-3.48	-3.17	<.001	0.63	-5.17	-5.07	Co-integration

[a] AIC: Akaike info criterion.

[b] SC: Schwarz criterion.

[c] Time series of monthly Baidu Index and incidence rate were co-integrated.
