# Supplementary material for: Association Between Cancer Incidence and Mortality in Web-Based Data in China: Infodemiology Study
Source: J Med Internet Res. 2019 Jan 29;21(1):e10677. doi: 10.2196/10677 (PMC6371071; doi:10.2196/10677)
Supplement: Multimedia Appendix 5 [file jmir_v21i1e10677_app5.pdf]

Table 4. Results of cointegration test of the two-time series of monthly Baidu indexes and mortality rates of cancers.

Cancer type	Unit root test for the residual series								Result [a]
	ADF	1% Level	5% Level	10% Level	*P* value	R^2	AIC value	SC value	
Lung cancer	-9.17	-4.09	-3.48	-3.17	<.001	0.56	-6.52	-6.42	Co-integration
Liver cancer	-8.69	-4.09	-3.48	-3.17	<.001	0.53	-6.70	-6.61	Co-integration
Stomach cancer	-7.94	-4.09	-3.48	-3.17	<.001	0.48	-8.66	-8.57	Co-integration
Colon and rectal cancer	-10.70	-4.09	-3.48	-3.17	<.001	0.63	-5.32	-5.22	Co-integration
Breast cancer	-12.66	-4.09	-3.48	-3.17	<.001	0.71	-6.16	-6.06	Co-integration
Esophageal cancer	-9.05	-4.09	-3.48	-3.17	<.001	0.55	-8.66	-8.57	Co-integration
Leukemia	-9.22	-4.09	-3.48	-3.17	<.001	0.56	-7.90	-7.81	Co-integration
Prostate cancer	-8.63	-4.09	-3.48	-3.17	<.001	0.53	-4.94	-4.84	Co-integration
Breast cancer	-9.89	-4.09	-3.48	-3.17	<.001	0.59	-6.21	-6.11	Co-integration
Cervical cancer	-10.17	-4.09	-3.48	-3.17	<.001	0.61	-7.22	-7.13	Co-integration
Pancreatic cancer	-12.53	-4.09	-3.48	-3.17	<.001	0.70	-4.64	-4.54	Co-integration
Uterine cancer	-8.28	-4.09	-3.48	-3.17	<.001	0.51	-7.22	-7.13	Co-integration
Non-Hodgkin lymphoma	-13.05	-4.09	-3.48	-3.17	<.001	0.72	-5.19	-5.09	Co-integration
Bladder cancer	-11.23	-4.09	-3.48	-3.17	<.001	0.65	-4.98	-4.89	Co-integration
Nasopharynx cancer	-9.11	-4.09	-3.48	-3.17	<.001	0.55	-8.05	-7.96	Co-integration
Lip and oral cavity cancer	-9.00	-4.09	-3.48	-3.17	<.001	0.55	-4.40	-4.30	Co-integration
Kidney cancer	-8.50	-4.09	-3.48	-3.17	<.001	0.52	-5.10	-5.00	Co-integration
Thyroid cancer	-8.38	-4.09	-3.48	-3.17	<.001	0.51	-5.95	-5.85	Co-integration
Squamous-cell carcinoma	NA	-4.09	-3.48	-3.17	<.001	NA	NA	NA	Co-integration
Larynx cancer	-12.83	-4.09	-3.48	-3.17	<.001	0.71	-5.14	-5.05	Co-integration
Ovarian cancer	-9.43	-4.09	-3.48	-3.17	<.001	0.57	-4.81	-4.71	Co-integration
Gallbladder and biliary tract cancer	-9.94	-4.09	-3.48	-3.17	<.001	0.60	-6.68	-6.58	Co-integration
Multiple myeloma	-13.17	-4.09	-3.48	-3.17	<.001	0.72	-3.96	-3.86	Co-integration
Malignant skin melanoma	-9.75	-4.09	-3.48	-3.17	<.001	0.59	-5.75	-5.65	Co-integration
Hodgkin lymphoma	-10.80	-4.09	-3.48	-3.17	<.001	0.64	-6.47	-6.37	Co-integration
Testicular cancer	-13.90	-4.09	-3.48	-3.17	<.001	0.74	-3.26	-3.17	Co-integration
Mesothelioma	-10.47	-4.09	-3.48	-3.17	<.001	0.63	-5.21	-5.11	Co-integration

[a] Time series of monthly Baidu Index and incidence rate were co-integrated.
